# Comparative genomics of downy mildews reveals potential adaptations to biotrophy

**DOI:** 10.1186/s12864-018-5214-8

**Published:** 2018-11-29

**Authors:** Kyle Fletcher, Steven J. Klosterman, Lida Derevnina, Frank Martin, Lien D. Bertier, Steven Koike, Sebastian Reyes-Chin-Wo, Beiquan Mou, Richard Michelmore

**Affiliations:** 1 The Genome Center, Genome and Biomedical Sciences Facility, University of California, 451 East Health Sciences Drive, Davis, CA 95616 USA; 2 United States Department of Agriculture, Agricultural Research Service, Salinas, CA 93905 USA; 3 UC Davis Cooperative Extension Monterey County, Salinas, CA 93901 USA; 4 Departments of Plant Sciences, Molecular & Cellular Biology, Medical Microbiology & Immunology, University of California, Davis, 95616 USA; 5 Present Address: The Sainsbury Laboratory, Norwich Research Park, Norwich, NR4 7UH UK; 6 Present Address: TriCal Diagnostics, Hollister, CA 95023 USA

**Keywords:** *Peronospora effusa*, *Peronospora farinosa*, Spinach downy mildew, Oomycete, Genomics, *Peronospora* lineage, Gene loss, Biotrophy

## Abstract

**Background:**

Spinach downy mildew caused by the oomycete *Peronospora effusa* is a significant burden on the expanding spinach production industry, especially for organic farms where synthetic fungicides cannot be deployed to control the pathogen. *P. effusa* is highly variable and 15 new races have been recognized in the past 30 years.

**Results:**

We virulence phenotyped, sequenced, and assembled two isolates of *P. effusa* from the Salinas Valley, California, U.S.A. that were identified as race 13 and 14. These assemblies are high quality in comparison to assemblies of other downy mildews having low total scaffold count (784 & 880), high contig N_50_s (48 kb & 52 kb), high BUSCO completion and low BUSCO duplication scores and share many syntenic blocks with *Phytophthora* species. Comparative analysis of four downy mildew and three *Phytophthora* species revealed parallel absences of genes encoding conserved domains linked to transporters, pathogenesis, and carbohydrate activity in the biotrophic species. Downy mildews surveyed that have lost the ability to produce zoospores have a common loss of flagella/motor and calcium domain encoding genes. Our phylogenomic data support multiple origins of downy mildews from hemibiotrophic progenitors and suggest that common gene losses in these downy mildews may be of genes involved in the necrotrophic stages of *Phytophthora* spp.

**Conclusions:**

We present a high-quality draft genome of *Peronospora effusa* that will serve as a reference for *Peronospora* spp. We identified several Pfam domains as under-represented in the downy mildews consistent with the loss of zoosporegenesis and necrotrophy. Phylogenomics provides further support for a polyphyletic origin of downy mildews.

**Electronic supplementary material:**

The online version of this article (10.1186/s12864-018-5214-8) contains supplementary material, which is available to authorized users.

## Background

Downy mildew diseases are caused by species of several genera of obligate biotrophic oomycetes and impact production of crops and ornamental plants worldwide [1]. The phylogenetic relationships of downy mildews to one another, as well as to closely related *Phytophthora* species (spp.), are unclear, with uncertainty as to how often the adaptation to obligate biotrophy has occurred [2–4]. Among the 19 downy mildew genera, *Peronospora* contains the highest number of species (~ 500) [1]. *Peronospora* spp. produce sexual oospores for survival in soil and plant debris, typical of oomycetes. However, unlike many oomycetes, the asexual sporangia of *Peronospora* germinate by forming a germ tube from the sporangia, rather than by releasing motile zoospores [1].

A prominent example of a destructive oomycete is *Peronospora effusa*, which causes spinach downy mildew; it is the most important pathogen of spinach globally [1, 5]. Like other pathogens in this genus, *P. effusa* has a narrow host range, only infecting spinach (*Spinacia oleracea*) [6, 7]. This pathogen has occasionally been incorrectly grouped with other *Peronospora* spp., such as *Peronospora schachtii* (causal agent of chard downy mildew) under the umbrella *Peronospora farinosa* [5, 6, 8–10], despite molecular data for distinct species [11, 12]. A formal rejection of the name *P. farinosa* (a.k.a. *Botrytis farinosa*) was recently proposed, in part because *P. farinosa* could not be associated with a type specimen [13].

Demand for and production of fresh market spinach is consistently high in the United States of America [14] and control of downy mildew is essential for sustainable production of spinach, particularly on organic farms. While synthetic fungicides have been effective in managing downy mildew diseases in conventional production [15], such fungicides are unavailable for organic spinach production. Therefore, the introduction of genes for resistance to downy mildew into spinach through breeding currently provides the most effective option for disease control for the burgeoning organic industry [15]. Based upon reactions of cultivars in the differential set used for screening isolates, six major loci for resistance to *P. effusa* have been proposed [8, 9]. Genome sequencing of *S. oleracea* identified 139 candidate resistance genes, five of which are closely linked to the *DM-1* gene, which confers resistance to *P. effusa* race (R) 6 [16].

New virulent races of *P. effusa* have appeared rapidly after the deployment of cultivars with new resistance genes, leading to a large increase in the number of races designated over the past decade [5]. Prior to the 1990s, only three *P. effusa* races had been described [17], to date 17 races have been denominated based on reactions to differentially susceptible or resistant lines [5, 8, 9, 18, 19]. The reason for the rapid appearance of the new races of *P. effusa* is not understood; the recent finding of oospores of *P. effusa* in ~ 16% of modern spinach seed lots has provided evidence for global movement of *P. effusa* on spinach seed [20] from seed production areas to fresh market production fields elsewhere. Sexual progeny from oospores will be highly variable because *P. effusa* is heterothallic [10, 21] and the global movement increases the potential for sexual recombination between novel combinations of isolates of *P. effusa.* While within-field genotypic diversity may be driven by asexual variation, the overall diversity of the species may be influenced by sexual recombination on broad temporal and geographic scales [19]. Oospores introduced into the fresh market production areas on spinach seed could introduce new combinations of virulence factors and contribute to the rapid demise of resistance genes.

Genome sequencing is now sufficiently inexpensive to permit the rapid sequencing and assembly of multiple isolates of small genome *Peronospora* spp. [22]. Recently the genomes of two *Peronospora tabacina* isolates were sequenced and shown to be compact and gene rich with fewer repeated sequences compared to other oomycetes [23]. While multiple species of several oomycete genera have been sequenced (e.g. *Phytophthora, Pythium, Saprolegnia, Aphanomyces*), only one genus (*Plasmopara*) of the obligately biotrophic downy mildews has had two species sequenced [3, 24, 25].

In this study, two isolates of *P. effusa* were collected from the field, virulence-phenotyped, and sequenced to produce high quality annotated assemblies comparable to other downy mildews and *Phytophthora* spp. *P. effusa*, like *P. tabacina*, has a small consensus genome size with few repeated sequences, although fewer gene models were identified. We contrasted these new assemblies and their gene models to other oomycetes and identified several domains that are under-represented in downy mildews with many orthologs missing when compared to *Phytophthora* spp. These data have implications to the loss of motile flagella and the necrotrophic mode of nutrition in downy mildews.

## Results

Two isolates of *P. effusa* were collected from commercial spinach production fields in Monterey County, California in 2012 and 2013 and their virulence phenotypes tested by inoculations onto the standard differential set of resistant spinach cultivars to provide race designations as previously described [8]. Cultivars Avenger, Lion (Solomon), and Pigeon were resistant to the 2012 isolate indicating that it was race 13 (R13), while cultivars Califlay, Whale, and Lion were resistant to the 2013 isolate indicating that it was race 14 (R14).

An additional isolate was collected from Monterey County in January 2016 to determine total nuclear DNA content. Flow cytometry revealed three peaks indicating nuclear DNA contents of 80 +/− 2 Mb, 149 +/− 9 Mb, and 300 +/− 18 Mb (Additional file 1). These results are consistent with the *P. effusa* isolate surveyed containing populations of nuclei within a coenocytic mycelium; a small proportion is approximately 80 Mb, while the majority are 149 Mb. The larger 300 Mb represents replicating 149 Mb nuclei.

### Read processing and assembly

Approximately 230 million 100 base-par (bp) paired-end reads were generated for both isolates that were reduced by 32% (R13) and 18% (R14) after quality and k-mer trimming. Read filtering identified approximately 58 and 97 million read pairs associated with other oomycete assemblies and 65 and 77 million un-associated read pairs for R13 and R14, respectively. Optimal k-mer lengths for highest output statistics were empirically found to be between 91 and 99 nt for both isolates. The top five BLASTn [26] filtered assemblies of R13 were assembled using k-mer sizes 92 to 96 and had scaffold N_50_s ranging from 20.9 k-base-pair (kb) to 23.8 kb with between 12 and 16 scaffolds over 100 kb with BUSCO [27] completeness (protist library) ranging from 94.9 to 97.9%. The top five R14 assemblies (k-mer sizes 92–95 + 97) had scaffold N_50_ ranges of 18,157 to 18,478 bp, with 18 to 23 scaffolds over 100 kb and BUSCO completeness ranging from 95.3 to 97.4%.

Post mitochondrial fragment filtering, pairwise merging of assemblies, from lowest scaffold count size to highest, and removal of fragments under 1 kb, the scaffold N_50_ of isolates R13 and R14 rose to 52.8 kb and 42.8 kb respectively and the highest BUSCO [27] score previously observed was retained in both isolates, although the duplication rate had increased. A single round of redundancy removal with Redundans [28] and Haplomerger2 [29] resulted in the final assemblies, with little redundancy detected by BUSCO. Complete statistics for the intermediate assemblies generated are provided in Additional file 2.

*P. effusa* R13 was assembled into 1475 contigs and 785 scaffolds. The contig N_50_ was 48.369 kb and the scaffold N_50_ was 72.2 kb. The assembly totaled 32.1 Mb, contained 0.26% gaps, and presented 68 scaffolds over 100 kb, three of which were over 250 kb. JELLYFISH [30] analysis produced an estimated haploid genome size of 44.1 Mega-base-pairs (Mb) with 26.6 Mb inferred as single copy (Additional file 3). BUSCO [27] reported a completeness of 97.8, 0.4% of complete BUSCOs were reported as duplicated and 2.2% reported as missing. There were no fragmented BUSCOs (Table 1).

**Table 1:** Comparative statistics of downy mildew genome assemblies and select *Phytophthora* assemblies

Genus	Species	Isolate/label	Scaffold N50 (kb)^a^	Scaffold count	Contig N50 (kb)	Contig Count	Assembly size (Mb)	Gaps (%)	Gene model count^b^	BUSCO				Reference
										Complete (%)	Duplicated (%)	Fragmented (%)	Missing (%)	
*Peronospora*	*effusa*	R13	72	784	48	1472	32.2	0.261	8607	97.8	0.4	0	2.2	This study
		R14	61	880	52	1275	30.8	0.564	8571	97	0	0.4	2.6	
	*tabacina*	968-J2	79	4016	11	10,799	63.1	27.351	11,310	94.9	29.5	3	2.1	[23]
		968-S26	61	3245	15	8552	55.3	19.089	10,707	94.9	29.1	3.4	1.7	
*Hyaloperonospora*	*arabidopsidis*	Emoy2	332	3044	43	10,401	78.9	10.224	14,321	96.6	4.7	2.6	0.8	[45]
*Psuedoperonospora*	*cubensis*	ASM25260v1	4	35,539	4	35,539	64.3	0	n/a	94	2.1	4.7	1.3	[82]
*Plasmopara*	*halstedii*	OS-Ph8–99-BlA4	**1546**	3162	16	25,359	75.3	11.322	15,469	97.4	0	1.7	0.9	[3]
	*viticola*	INRA-PV221	181	1883	49	3995	74.7	2.83	n/a	95.7	4.7	1.7	2.6	[24]
		JL-7-2	172	2165	14	23,193	101.2	16.712	n/a (17,014)	84.6	8.1	8.5	6.9	[25]
*Sclerospora*	*graminicola*	UoM-SG-P.1	18	26,786	16	28,799	299.9	0.29	n/a (38,120)	86.4	12.0	4.3	9.3	[102]
*Phytophthora*	*infestans*	T30–4	**1589**	4921	44	18,288	228.5	16.806	17,797	97	3	1.3	1.7	[103]
	*ramorum*	ASM14973v1	308	2576	48	7589	66.7	18.346	15,605	97.4	3	1.7	0.9	[104]
	*sojae*	Physo3	**7609**	83	386	863	82.6	3.959	26,489	99.5	3.8	0	0.5	[104]

^a^Numbers over 1000 kb (1 Mb) are highlighted with bold typeface

^b^n/a as gene models are not available from a public resource for download. Bracketed numbers are reported from the reference article

*P. effusa* R14 was assembled in to 1275 contigs and 880 scaffolds. The contig N_50_ was 51.71 kb and the scaffold N_50_ was 61.4 kb. The assembly totaled 30.8 Mb, contained 0.56% gaps and had 47 scaffolds over 100 kb, one of which was over 250 kb. JELLYFISH analysis produced an estimated haploid genome size of 41.2 Mb with 27.9 Mb inferred as single copy (Additional file 3). BUSCO reported a completeness of 97.0%, with no duplicated predictions and 0.4% fragmented predictions. The remaining 2.6% BUSCOs were reported as missing (Table 1).

### Assembly quality

**Fig. 1:**
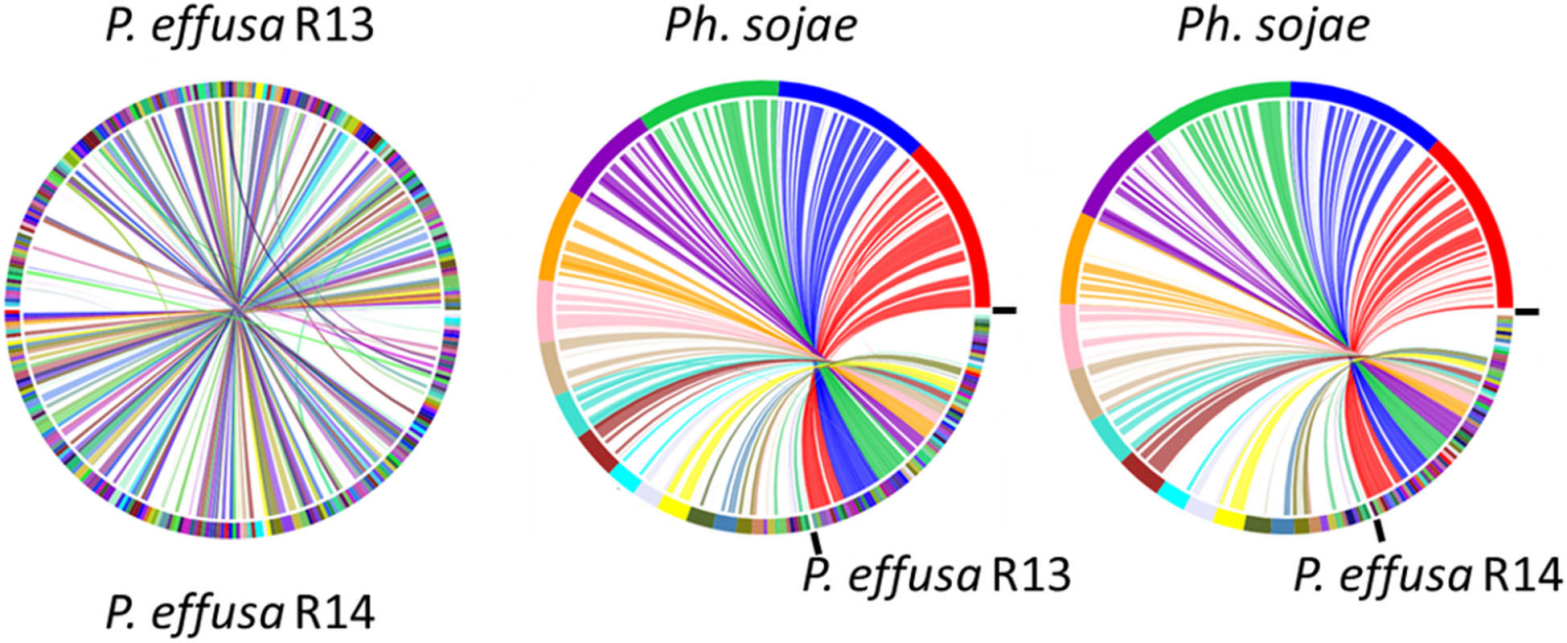
SyMap plots of *P. effusa* R13 aligned against *P. effusa* R14, *P. sojae* v3.0 against *P. effusa* R13 and *P. sojae* v3.0 against *P. effusa* R14. The plots aligned against *P. sojae* are scaled so the area of the plot occupied by *P. sojae* scaffolds is scaled to the size of the *P. effusa* sequences. No scaling is applied to the *P. effusa* cross isolate comparison

SyMap [31] plots showed that the two *P. effusa* assemblies had a high degree of collinearity although they are highly fragmented (Fig. 1). There were 237 and 214 scaffolds over 50 kb for R13 and R14, respectively; inter-isolate alignments were identified for 141 R13 and 134 R14 of these scaffolds. When compared to *P. sojae*, 177 R13 and 167 R14 scaffolds over 50 kb could be aligned with 23 *P. sojae* scaffolds over 50 kb.

### Repeat masking and LTR analysis

Repeat libraries for both isolates were generated using RepeatModeler [32], identifying 98 elements in R13 and 88 in R14. In both isolates most elements identified were Long Terminal Repeat (LTR) Retrotransposons (Additional file 4). The LTR profiles of the two isolates of *P. effusa* were similar to one another, although the density profile indicates that isolate R14 may have more similar Copia elements than R13 (Additional file 5).

### Annotation

Initial SNAP [33] hidden Markov model (HMM) training with MAKER [34] resulted in 7853 gene predictions for R13. Subsequent runs with intermediate SNAP bootstrapping produced predictions ranging from 6493 to 9601 models, the mean residue length ranged from 407 to 482 amino acids. Equivalent bootstrapping of the same HMM run on *P. effusa* R14 produced predictions ranging from 6691 to 9034 gene models, the mean residue length ranged from 407 to 476 amino acids (Additional file 6). Further assessment of each individual unfiltered run showed mean BLAST [26] scores back to the training data ranging between 569 to 671 for R13 and 603 to 678 for R14 (Additional file 6). When orthology analysis was performed for each run against the training data, the number of orthogroups detected varied from 4803 to 7117 for R13 and 5363 to 7073 for R14 (Additional file 6). The mean e-value for Pfam [35] domains, scoring under 1e^− 5^, of individual MAKER runs ranged from 1.99 e^− 7^ to 2.20 e^− 7^ for R13 and 1.99 e^− 7^ to 2.23 e^− 7^ for R14 (Additional file 6). For R13 the MAKER run with the highest mean BLAST score, second highest orthogroup count, and lowest mean Pfam e-value was considered the best. For R14, the best annotation set scored the highest mean BLAST score, highest orthogroup count, and lowest mean Pfam e-value (Additional file 6). Investigating gene models at unique loci from alternative MAKER runs did not produce a high scoring set of gene models; therefore, integrating unique models from multiple runs was not performed.

Independent translation of the entire genome of both isolates, followed by effector identification through a combination of HMM-profiling and string searches (Additional file 7) resulted in a total of 148 and 137 putative single open reading frame (ORF) effectors. Intersecting these loci with MAKER predictions identified 41 and 43 previously unpredicted loci for each isolate (Additional file 8). Putative effectors were manually curated in cases of overlapping or flanking ORFs. Reconciliation of these cases resulted in three multi-exonic effectors for both isolates, in every case the first exon contained a signal peptide, RxLR motif, and EER motif as well as possibly more than one WY domain. The second exon always contained at least one WY domain. These were added with the other ORF predictions to their respective annotated gene sets (Additional file 9). The effector prediction pipeline was run on the final gene sets, producing counts of 113 and 107 putative effectors for the draft assemblies of R13 and R14 respectively (Table 2).

**Table 2:** Putative effectors identified through regular expression and HMMs

Category	R13	R14
RxLR-[DE][DE][ER]	33	34
[GHQ]xLR-[DE][DE][ER]	7	7
RxL[GKQ]-[DE][DE][ER]	14	12
RxLR-WY	12	12
[GHQ]xLR-WY	0	0
RxL[GKQ]-WY	1	0
RxLR-[DE][DE][ER]-WY	24	20
[GHQ]xLR-[DE][DE][ER]-WY	1	0
RxL[GKQ]-[DE][DE][ER]-WY	1	2
Total	93	87
CRN (Secreted)	20 (8)	20 (5)

### Comparative analysis

Gene models encoding putative pathogenicity domains were identified through InterProScan for each assembly (Table 3). T-tests indicated that the frequencies of gene models encoding several of these domain types were significantly different between *Phytophthora* spp. and downy mildews, as was the total frequency of pathogenicity related models. The frequency of pathogenicity-associated gene models in *Phytophthora* spp. ranged between 0.026 to 0.036, while all downy mildews had lower incidences ranging from 0.013 to 0.017. The two isolates of *P. effusa* had the highest incidence of pathogenicity genes of the downy mildews analyzed.

**Table 3:** Putative pathogenicity domain encoding genes of *P. effusa* and related oomycetes

	*Peronospora*				*Hyaloperonospora arabidopsidis*	*Plasmopara halstedii*	*Phytophthora*			*Phytophthora* spp. vs. downy mildews	*Peronospora* lineage vs. *Phytophthora* spp. + *P. halstedii*
	*effusa*		*tabacina*								
	R13	R14	J2	S26			*sojae*	*infestans*	*ramorum*	t-test	t-test
Serine protease	14	13	21	12	12	27	42	37	36		
Aspartic protease	11	9	17	7	15	26	147	20	116	< 0.05	
Cysteine protease	16	17	18	17	16	20	28	27	30		
Metalloprotease	16	18	14	16	25	17	38	24	26		
Kazal-like serine protease inhibitor	1	1	2	2	7	17	45	35	17	< 0.01	< 0.001
Cystatin-like cysteine protease inhibitor	1	1	1	1	1	3	4	6	4	< 0.05	< 0.01
Cutinase	0	0	0	0	2	2	15	4	4	< 0.01	< 0.05
Pectin lyase	11	13	6	8	22	19	122	100	82	< 0.001	< 0.05
CAP domain	39	42	45	48	38	72	155	112	104	< 0.01	
NPP1-like	9	10	17	14	21	19	80	28	62	< 0.05	< 0.05
Elicitin-like	16	15	12	8	20	20	77	54	61	< 0.001	< 0.05
Jacalin	10	10	7	7	10	20	31	22	27		< 0.05
Frequency	0.0168	0.0173	0.0141	0.0130	0.0132	0.0167	0.0295	0.0262	0.0361	< 0.001	< 0.05

Analysis (chi-squared) of Pfam domains revealed 96 as significantly enriched or depleted relative to their expected distributions, scoring below the Bonferroni adjusted e-value of 1.33 e^− 5^ (Additional file 10) in at least one of the four multi-species comparisons performed (Table 4). Of these, six were enriched but were excluded because they had a skewed over-representation in *P. tabacina* (possibly due to under assembly of the genomes; see section on K-mer analysis and heterozygosity below) compared to *P. effusa* and *Hyaloperonospora arabidopsidis*; excluding *P. tabacina* resulted in insignificant scores for these six. The 90 remaining Pfam domains were all indicated as depleted in the *Peronospora* lineage made up of *P. effusa*, *P. tabacina* and *H. arabidopsidis*, when compared to *Phytophthora* spp. (Table 4). In addition, 64 of the 90 domains were also under-represented in *Plasmopara halstedii* including 24 of which could be grouped as phytopathology, transporter and carbohydrate associated domains. When *P. halstedii,* the only downy mildew analyzed that has motile flagella, was grouped with the *Phytophthora* spp., 26 domains obtained a more significant score including 14 in the classes: flagella apparatus and calcium associated domains. These five classes contained 42% (38/90) of the domains detected as significantly depleted; the other 58% could not be assigned to one of these classes (Additional file 10).

**Table 4:** Chi-square results of Pfam domain representation in gene models of 9 oomycete assemblies

Pfam	*P. effusa* vs all	*Peronospora* spp. vs. All	*Peronospora *lineage vs. all	Downy mildew vs. *Phytophthora* spp.	Pfam title	Category
PF11051		8.34e^−10^	**6.11e** ^**−10**^	8.62e^−10^	Mannosyltransferase	Carbohydrate associated
PF00232				**7.81e** ^**−8**^	Glycosyl hydrolase family 1	
PF00295		3.66e^−6^	7.04e^−8^	**4.71e** ^**−11**^	Glycoside hydrolase family 28	
PF00933		1.20e^−5^	1.70e^−6^	**1.69e** ^**− 8**^	Glycosyl hydrolase family 3 N terminal domain	
PF01762				**3.93e** ^**−6**^	Galactosyltransferase	
PF01915			3.19e^− 6^	**2.45e** ^**− 8**^	Glycoside hydrolase family 3	
PF00612		2.17e^−6^	**2.88e** ^**−7**^		IQ calmodulin binding motif	Calcium associated
PF13202		4.68e^−6^	**9.71e** ^**−9**^	9.92e^−7^	EF hand	
PF13499		3.70e^−8^	**4.59e** ^**−10**^	9.75e^− 8^	EF hand	
PF13833			**1.13e** ^**−7**^		EF hand	
PF00225		1.16e^−7^	**7.33e** ^**−10**^	2.75e^−6^	Kinesin motor domain	Flagella / Motor associated
PF07728		9.14e^−6^	**6.35e** ^**−8**^		ATPases associated; dynein related subfamily	
PF03028		6.28e^− 9^	**4.15e** ^**−12**^	9.94e^−8^	Dynein heavy chain and region D6 of dynein motor	
PF08385			**2.85e** ^**−6**^		Dynein heavy chain	
PF08393		1.94e^−9^	**1.51e** ^**−13**^	3.36e^− 9^	Dynein heavy chain, N-terminal region 2	
PF12774		8.43e^−9^	**1.23e** ^**−12**^	4.65e^−8^	ATPases associated; P-loop containing dynein motor region	
PF12775		2.04e^−8^	**4.35e** ^**− 12**^	2.18e^−7^	ATPases associated; P-loop containing dynein motor region	
PF12777		3.49e^−9^	**3.50e** ^**−13**^	9.69e^− 9^	Microtubule-binding stalk of dynein motor	
PF12780		2.21e^−7^	**6.99e** ^**−11**^	1.49e^−6^	ATPases associated; P-loop containing dynein motor region-D4	
PF12781		2.74e^−8^	**6.61e** ^**−12**^	3.62e^−7^	ATPases associated; P-loop containing dynein motor region-D5	
PF00050		1.46e^−8^	**2.47e** ^**−9**^	1.06e^−7^	Kazal-type serine protease	Phytopathology associated
PF00734			**4.92e** ^**−6**^	1.14e^−5^	Fungal cellulose binding domain	
PF07648		9.31e^−8^	**4.11e** ^**−10**^	2.77e^−08^	Kazal domain	
PF00544		5.44e^−7^	1.88e^−6^	**9.83e** ^**− 10**^	Pectate lyase	
PF00964		1.17e^−8^	5.92e^−10^	**1.87e** ^**−12**^	Elicitin	
PF02902		2.59e^−10^	1.53e^−14^	**1.62e** ^**−20**^	ULP1 protease family, C-terminal catalytic domain	
PF03211		3.68e^−13^	1.21e^−13^	**6.77e** ^**−19**^	Pectate lyase	
PF05630		3.81e^−09^	3.88e^−9^	**9.46e** ^**−12**^	Necrosis inducing protein	
PF09461				**5.22e** ^**−6**^	Phytotoxin PcF	
PF16810	6.38e^−10^	4.13e^−32^	6.61e^−45^	**1.08e** ^**−64**^	RxLR phytopathogen effector protein.	
PF00083		3e^−7^	**1e** ^**−10**^	3e^−6^	Sugar (and other) transporter	Transporter associated
PF00520	1.03e^−6^	2.86e^−14^	**3.15e** ^**−21**^	7.97e^−21^	Ion channel family	
PF00005	3.84e^−6^	1.32e^−19^	9.28e^−23^	**5.70e** ^**−32**^	ABC transporter	
PF00664		2.11e^−9^	8.42e-11	**2.34e-14**	Transmembrane domain of ABC transporters	
PF01061		2.36e^−17^	2.11e-20	**3.45e-29**	ATP-binding cassette transporter	
PF03083		1.63e^−7^	1.88e-10	**2.16e-14**	Sugar efflux transporter for intercellular exchange	
PF03092				**5.98e** ^**−7**^	BT1 family	
PF06422		3.41e^−10^	5.84e-10	**1.37e-13**	CDR ABC transporter	

The bold score for each Pfam domain is the best p-value obtained from all four comparisons

**Fig. 2:**
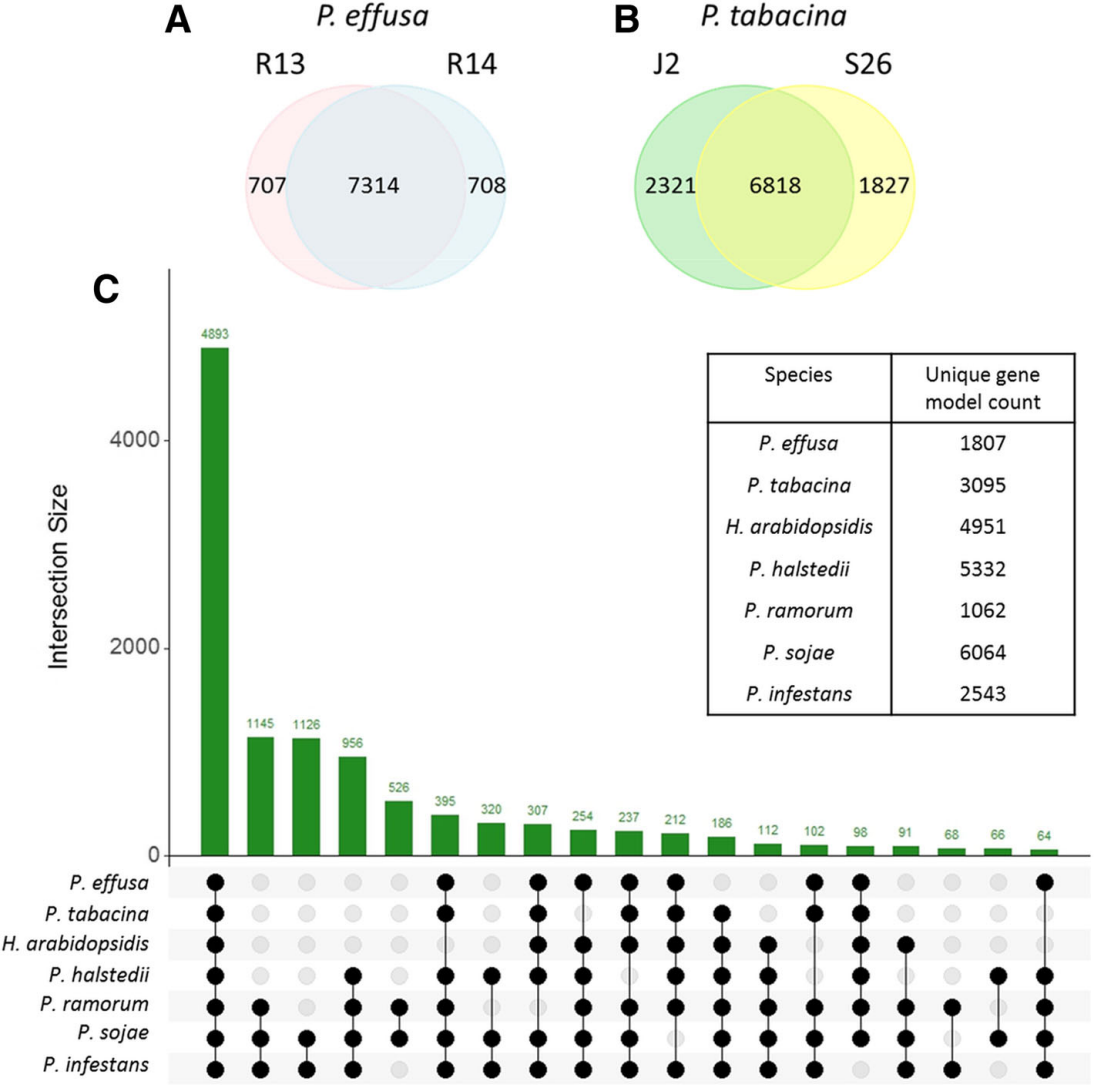
Orthology analysis of oomycete gene models. **a** Venn diagram depicting the number of orthogroups shared between two *P. effusa* isolates and unique gene counts. **b** Venn diagram depicting the number of orthogroups shared between two *P. tabacina* and unique gene counts. **c** UpsetR plot demonstrating the number of orthogroups shared by sets of oomycete species. The intersection size is the number of orthogroups and the black dots on the x-axis represent whether the orthogroups are present or absent in that set. For instance, the first bar demonstrates that 4893 orthogroups are shared among all oomycetes, while the second demonstrated that 1145 are shared among *Phytophthora* spp. and are absent in the downy mildews. Only intersections over 60 orthogroups are depicted. The table inset shows how many gene models are unique to each species and include models not assigned to orthogroups and models included in orthogroups made up from a single species

Orthology analysis between the two isolates of *P. effusa* identified 7314 overlapping orthogroups, of which 6833 were single copy. Nine orthogroups, containing 54 genes and a further 653 singletons unassigned to orthogroups were found exclusive to R13, while 10 orthogroups, containing 70 genes and a further 638 singletons were exclusive to R14. These isolate-specific genes represented 8% of the total predicted genes (Fig. 2a). Reads were mapped between isolates to determine the inter isolate coverage of coding regions and to determine if SNPs/short-indels could be detected in genes to determine why the proteins were inferred as absent. Mapping reads of R14 to R13 revealed 40 R13 genes that had low coverage, one of which encoded an RxLR effector. Seven genes contained indels within their coding regions. Additionally, 28 genes, including one encoding a necrosis inducing protein (NPP1), had SNPs which introduced a premature stop codon. SNPs in 14 genes resulted in the loss of their start codon and the loss of the stop codon in 22 genes, relative to the genes predicted in R13. When R13 reads were mapped to R14, ten genes had low coverage, including two RxLR effectors, which had a single residue difference between one another. Fifteen genes, including one RxLR contained indels within their coding regions. Additionally, 31 genes, including one CRN, had SNPs which introduced premature stop codons, 22 genes, including one RxLR and a gene encoding a protein kinase domain, had SNPs which caused the loss of the stop codon and nine lost their start codon, relative to the genes predicted for R14. These proteins, especially those with putative virulence/necrotic function may be useful as diagnostic markers for each race (Additional file 11). Repeating the analysis on the two previously sequenced isolates of *P. tabacina* [23] revealed 6818 overlapping orthogroups, 3869 of which were single copy and 19% of the gene models reported as unique to either of the isolates (Fig. 2b).

Species-level orthology analysis were performed by combining the gene models predicted for both isolates of each *Peronospora* spp. and comparing these with predictions from single isolates of *H. arabidopsidis*, *P. halstedii*, *Phytophthora infestans, Phytophthora sojae* and *Phytophthora ramorum*. This identified 12,835 orthogroups (Fig. 2c). Of these, 4893 were ubiquitous to all species, 2865 were present in at least two *Phytophthora* spp. and exclusive to this clade, 956 orthogroups were ubiquitous and exclusive to *Phytophthora* spp. and *P. halstedii*, 237 were ubiquitous to all assemblies except *P. halstedii*, 395 were ubiquitous to all assemblies except *H. arabidopsidis,* and 112 excluded both *Peronospora* spp. (Fig. 2c). Only 43 orthogroups, containing 154 gene models were unique to *Peronospora* spp. (Additional file 12). *P. effusa* scored its highest pairwise orthology coefficient with *P. tabacina* (0.82) followed by with *H. arabidopsidis* (0.77), *P. halstedii* (0.68) and *Phytophthora* spp. (0.54 to 0.58). The orthology coefficient showed a general trend grouping *Phytophthora* spp. (0.72 to 0.86) and *Peronospora* spp. plus *H. arabidopsidis* (0.76–0.82); however, *P. halstedii* did not group well with either, scoring 0.65 to 0.67 with *Phytophthora* spp. and 0.66 to 0.68 with *Peronospora* spp./*H. arabidopsidis*. *P. effusa* shared more orthologous groups, than *P. tabacina* or *H. arabidopsidis*, with all *Phytophthora* spp. and *P. halstedii* and consistently scored a higher orthology coefficient than *P. tabacina* and *H. arabidopsidis,* when compared to all three *Phytophthora* spp. and *P. halstedii* (Table 5). These higher orthology scores for *P. effusa* are consistent with high quality of the gene model predictions for this species.

**Table 5:** Pairwise overlaps of orthology groups (top right) and calculated orthology coefficients (bottom left bold and bracketed on intersecting diagonal) of publicly available gene models of downy mildew genome assemblies and select *Phytophthora* spp.

	*P. effusa* ^a^	*P. tabacina* ^a^	*H. arabidopsidis*	*P. halstedii*	*P. ramorum*	*P. sojae*	*P. infestans*
*P. effusa* ^a^	7430 **(1)**	6646	6425	6572	6557	6860	6882
*P. tabacina* ^a^	**0.82**	7209 **(1)**	6283	6421	6378	6680	6755
*H. arabidopsidis*	**0.77**	**0.76**	7211 **(1)**	6388	6365	6638	6668
*P. halstedii*	**0.68**	**0.67**	**0.66**	8578 **(1)**	7469	8020	8055
*P. ramorum*	**0.58**	**0.57**	**0.56**	**0.65**	9963 **(1)**	9436	9016
*P. sojae*	**0.54**	**0.52**	**0.52**	**0.64**	**0.76**	11,798 **(1)**	10,711
*P. infestans*	**0.57**	**0.56**	**0.55**	**0.67**	**0.72**	**0.86**	11,272 **(1)**

^a^Two isolates used in analysis

**Fig. 3:**
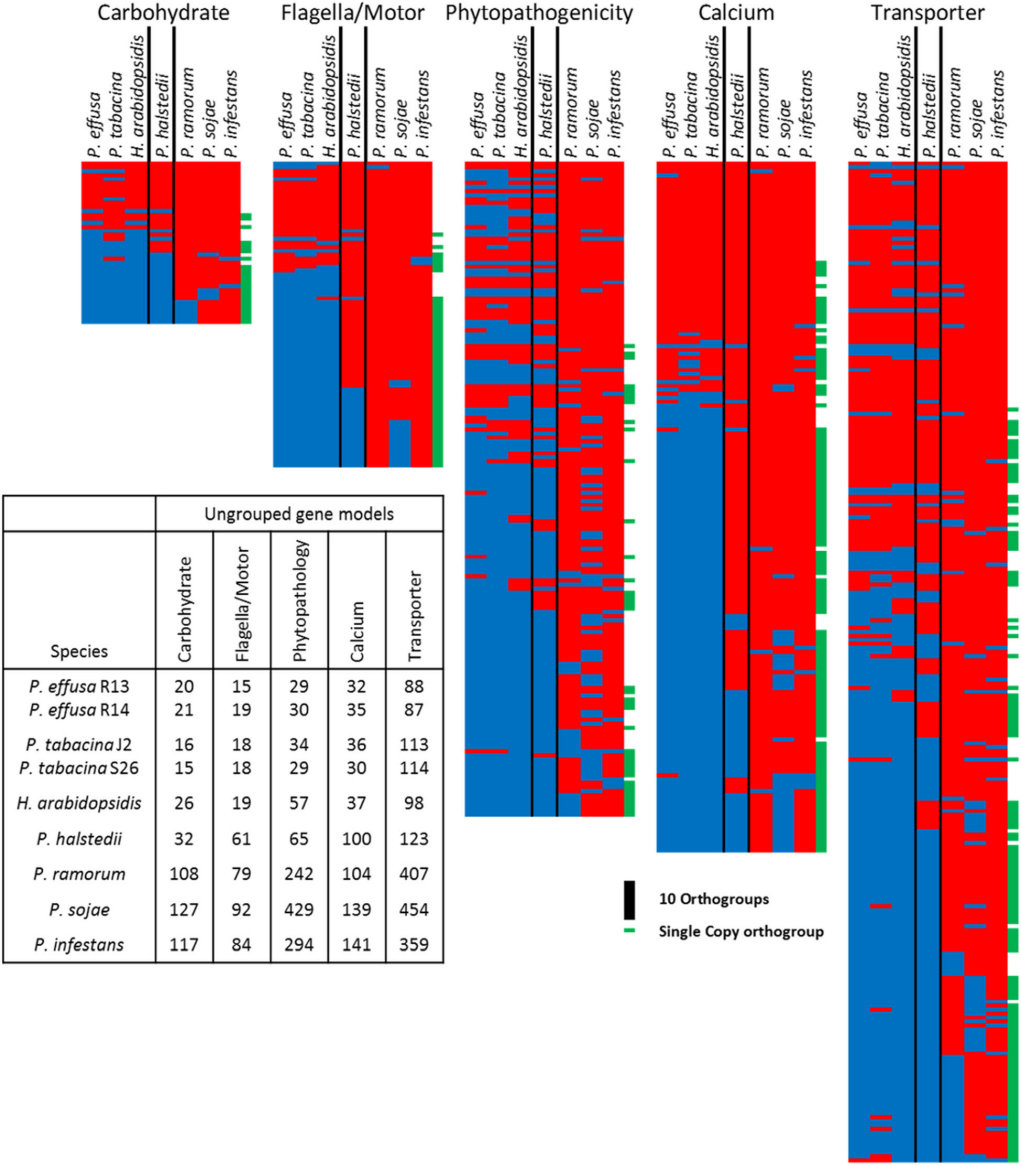
Heat map showing the distribution of orthogroups where a Pfam domain, found to be depleted in downy mildews, is detected as encoded by at least one gene model of that orthogroup. Red indicates the orthogroup contains at least one model from the species, blue indicates that no model is detected from the species. Green tabs on the right of the row indicates that the orthogroup contains a maximum of one gene from each species except for *P. effusa* and *P. tabacina* where two models were permitted as two isolates were combined in the analysis. *P. halstedii* is bordered to highlight that it does not have the same loss pattern exhibited by other downy mildew species across all categories. The inlayed table indicates the number of gene models that encode an under-represented Pfam domain, but were not assigned to an orthogroup

There were 709 orthogroups containing at least one protein encoding a Pfam domain inferred as under-represented in *Peronospora* species/*H. arabidopsidis* and grouped under one of the previously defined categories (Table 4). These were visualized to investigate the absence of the orthologs in downy mildew species (Fig. 3). While a core component of each orthogroup, typically the high gene number groups in all categories except phytopathogenicity, are retained across all oomycetes tested (Table 6), the majority of orthogroups for each category have missing orthologs for *Peronospora* sp. and *H. arabidopsidis*. When these are grouped with *P. halstedii* over 50% of orthogroups have detectable orthologs for downy mildew species in every category except phytopathogenicity, with a much larger fraction of motor and calcium associated domain encoding orthologs being identified than carbohydrate and transporter orthologs in *P. halstedii* (Table 6). The counts and IDs of the proteins contained within each orthogroup are supplied as Additional file 13.

**Table 6:** Presence of orthologs encoding domains under-represented in downy mildews

	All		> 1 *Phytophthora* species		> 1 downy mildew		> 1 *Peronospora* lineage species	
	# Orthogroups	# Single Copy Orthogroups	# Orthogroups	# Single Copy Orthogroups	# Orthogroups	# Single Copy Orthogroups	# Orthogroups	# Single Copy Orthogroups
Calcium	174	127	174 (100%)	127 (100%)	133 (76.4%)	92 (72.4%)	62 (35.6%)	29 (22.8%)
Carbohydrate	41	22	41 (100%)	22 (100%)	23 (56.1%)	7 (31.8%)	20 (48.9%)	4 (18.2%)
Flagella/Motor	77	50	77 (100%)	55 (100%)	57 (74.0%)	30 (60.0%)	27 (35.1%)	8 (16.0%)
Phytopathogenicity	165	41	163 (98.8%)	40 (98.6%)	75 (45.5%)	21 (51.2%)	61 (37.0%)	15 (36.6%)
Transporter	252	127	251 (99.6%)	126 (99.2)	144 (57.1%)	59 (46.5%)	117 (46.4%)	43 (33.9%)

Orthologous groups associated with nitrogen and sulphur assimilation were investigated (Table 7). Neither of the two nitrate reductase paralogs present in all three *Phytophthora* spp. were detected in the six assemblies of the four downy mildew species analyzed. The nitrate transporter orthogroup contained 4 to 6 paralogs for each *Phytophthora* spp. with 1 to 2 homologs identified for each *Peronospora* spp./*H. arabidopsidis*; no ortholog was identified from *P. halstedii*. Sulfite reductase genes were identified in one isolate of *P. tabacina* and both *P. effusa* assemblies but were not detected in *H. arabidopsidis* and *P. halstedii*. Orthologs of other nitrogen and sulphur assimilation associated enzymes were nearly ubiquitous in the tested assemblies, except no orthologs of glutamate synthase were detected in one of the isolates of *P. tabacina* and no orthologs of glutamine synthetase were detected in *P. halstedii*.

**Table 7:** Orthology assignment of nitrogen & sulphur assimilation enzymes described previously. Orthogroups are separated by borders

Putative function	*P. infestans*	*P. ramorum*	*P. sojae*	*H. arabidopsidis*	*P. tabacina* 968-J2	*P. tabacina* 968-S26	*P. effusa* R13	*P. effusa* R14	*P. halstedii*
Nitrate reductase	XP_002900554.1	Phyra76696	XP_009533168.1	–	–	–	–	–	–
	XP_002900553.1	Phyra71442	XP_009533167.1	–	–	–	–	–	–
Nitrate transporter	XP_002900550.1 XP_002900551.1 XP_002900552.1 XP_002903614.1	Phyra43555 Phyra43556 Phyra76698 Phyra76702 Phyra76703	XP_009526093.1 XP_009533166.1 XP_009533172.1 XP_009533173.1 XP_009533174.1 XP_009535913.1	HpaP804258	Ptab1_000523.1 Ptab1_001793.1	Ptab2_000415.1 Ptab2_023789.1	PeffR13_006272-RA	PeffR14_007341-RA	–
Glutamine synthetase	XP_002899289.1 XP_002899288.1	Phyra72153 Phyra72154	XP_009537735.1 XP_009537736.1	HpaP802420	Ptab1_007691.1	Ptab2_018669.1	PeffR13_007392-RA	PeffR14_006695-RA	–
Glutamate synthase (NADH)	XP_002904413.1	Phyra72102	XP_009528137.1	HpaP805196	–	Ptab2_009166.1	PeffR13_005711-RA	PeffR14_000994-RA	Phal12782
Glutamate synthase (Ferridoxin)	XP_002901469.1 XP_002897669.1	Phyra78125	XP_009535431.1	HpaP812981	Ptab1_006627.1	Ptab2_001634.1	PeffR13_005438-RA	PeffR14_005090-RA	Phal04802
Glutamate dehydrogenase	XP_002904619.1	Phyra71959	XP_009534337.1	HpaP805610 HpaP806677	Ptab1_013770.1 Ptab1_014043.1	Ptab2_016507.1	PeffR13_004916-RA	PeffR14_007520-RA	Phal12692
ATP sulfurylase Adenylylsulfate kinase Pyrophosphatase	XP_002907034.1	Phyra79353	XP_009527256.1	HpaP813786	Ptab1_021733.1	Ptab2_020487.1	PeffR13_005995-RA	PeffR14_007707-RA	Phal00799
Phosphoadenosine phosphosulfate reductase	XP_002905356.1	Phyra74880	XP_009520480.1	HpaP809449	Ptab1_009492.1	Ptab2_001511.1	PeffR13_006965-RA	PeffR14_006997-RA	Phal12096
Cysteine synthetase	XP_002900123.1 XP_002900124.1	Phyra71224 Phyra71225	XP_009530251.1 XP_009530252.1	HpaP814750	Ptab1_008049.1	Ptab2_003698.1	PeffR13_004177-RA	PeffR14_003698-RA	Phal08105
Sulfite reductase	XP_002997199.1	Phyra81878	XP_009516220.1	–	–	Ptab2_003268.1 Ptab2_013985.1	PeffR13_005098-RA	PeffR14_004743-RA	–
	XP_002896336.1	Phyra81882	XP_009516225.1	–	–	Ptab2_000240.1	PeffR13_005095-RA	PeffR14_004746-RA	–

Note added in proof. P. effusa R13 proteins can be queried from GenBank by substituting the string PeffR13 with DD237 and removing the -RA suffix. For P. effusa R14 replace PeffR14 with DD238 and remove the -RA suffix

### K-mer analysis and heterozygosity

**Fig. 4:**
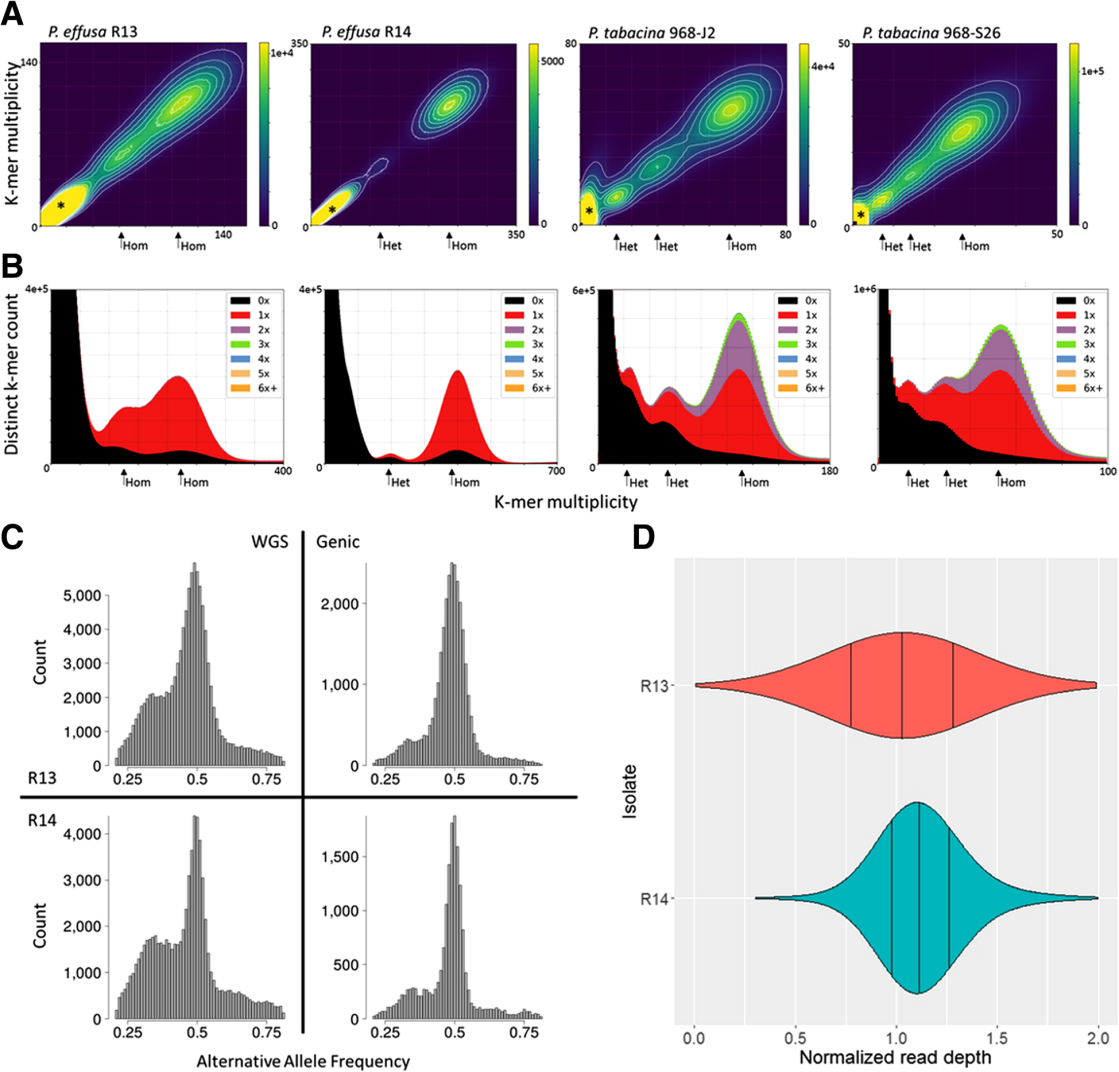
K-mer and read mapping overview. **a** KAT density plots demonstrating the 21-mer multiplicity of read 1 (x axis) against read 2 (y axis) for *P. effusa* R13, *P. effusa* R14*, P. tabacina* J2 and *P. tabacina* S26. Approximate locations of homozygous and heterozygous 21-mer are labeled on the x-axis. **b** KAT spectra-cn plots of the 21-mer multiplicity of the read set (x axis) against their respective assembly (y axis) for *P. effusa* R13, *P. effusa* R14, *P. tabacina* J2 and *P. tabacina* S26. Black areas under the peaks represent 21-mers present in the reads, absent in the assembly, red indicates 21-mers are present once in the assembly, purple 21-mers are present twice, green thrice. **c** Frequency of reads supporting the alternative allele over the entire assembly and gene space for *P. effusa* R13 and *P. effusa* R14. The allele frequency is cut-off at 0.2 and 0.8 on the x-axis of all plots. **d** Normalized read depth of every gene predicted in both *P. effusa* R13 and *P. effusa* R14. The height of each plot indicates the number of gene models at the normalized coverage displayed on the x axis

KAT density plots [36] were made to investigate heterozygosity in *P. effusa*. Two clusters of 21-mers are visible in both plots in addition to the many low-frequency 21-mers due to sequencing errors and contaminants (Fig. 4a). R14 has strong homozygous k-mer signal and a weak heterozygous signal at half coverage. R13 has strong homozygous k-mer signal and a significant signal at higher than half coverage. The same analysis of two *P. tabacina* isolates [23] detected strong homozygous k-mer signal and two heterozygous signals at half and quarter coverage consistent with the presence of multiple distinct haplotypes due to polyploidy, heterokaryosis, or mixtures for both isolates (Fig. 4a).

Spectra-cn plots that are also generated by KAT [36] were used to investigate the frequencies of k-mers in common between the read sets and assemblies of *P. effusa*. R14 contains one significant peak with the majority of 21-mers being represented once in the assembly, consistent with a high-quality assembly of a predominantly homozygous organism (Fig. 4b). The majority of 21-mers of the R13 read set were represented once, regardless of the k-mer frequency in the read set; this indicates that neither cluster of k-mers in R13 was heterozygous because most of the k-mers in the lower coverage peak were incorporated in the assembly rather than the anticipated proportion (50%) of the k-mers being absent. Therefore, the assembly of R13 seems to be of high quality but these k-mer plots are not consistent with a simple diploid genome. In contrast, spectra-cn plots of *P. tabacina* confirmed that both isolates contained three clusters of k-mers with a high proportion of k-mers from the first peaks absent in the assembly, approximately half the k-mers absent from the second peaks, and a small fraction absent from the homozygous peaks. Both isolates of *P. tabacina* had high levels of k-mer duplication; a significant proportion in the homozygous peak was represented twice as much as expected in the assembly indicating that both *P. tabacina* assemblies are under-assembled (Fig. 4b).

Reads were mapped back to the respective assemblies to identify single nucleotide polymorphisms (SNPs) and investigate the frequency of reads supporting the alternative allele in *P. effusa*. In R13, 106,714 heterozygous SNP sites were identified and 74,690 in R14, indicating 0.33 and 0.24% heterozygosity for R13 and R14, respectively (Fig. 4c). Plots of the frequency of reads supporting the alternative allele at each SNP revealed a clear peak at 0.5 in both isolates as expected in a diploid; however, a smaller second peak was present for both isolates at ~ 0.33. When only SNPs in genes were considered, the SNP count was reduced to 31,041 and 17,943 inferring a 0.23 and 0.13% heterozygosity in the predicted gene space of R13 and R14, respectively. Plots of the frequency of reads supporting the alternative allele of genic SNPs retained the peak at 0.5, although the peak at 0.33 was greatly reduced, containing ~ 5.7% (R13) to 8.6% (R14) of the genic SNPs (Fig. 4c). Collectively, these data indicate that the allele frequencies of numerous SNPs in *P. effusa* are not consistent with the 1:1 ratio expected in a diploid organism.

The normalized coverage of each predicted gene in R13 was more variable than in R14 (Fig. 4d). While the majority of the ~ 8600 predicted genes had around 1 to 1.2x normalized coverage in both isolates, 851 gene models in R13 had 0.6 to 0.8x coverage; in contrast, only 67 genes in R14 had 0.6 to 0.8x coverage. There was no significant deviation in representation of the 513 Pfam domains encoded by these genes.

### Phylogenetics

**Fig. 5:**
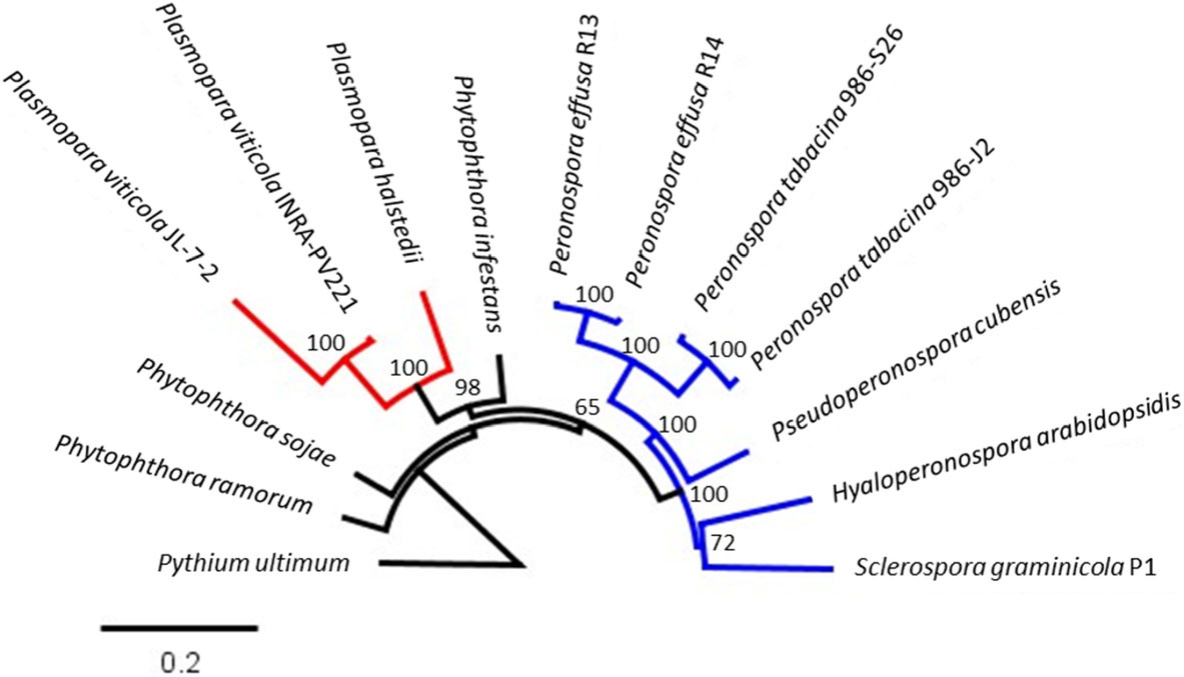
Maximum likelihood phylogeny of 49 BUSCO gene models, identified as conserved as a single copy gene across all assemblies surveyed. Node labels indicate the score after 1000 bootstraps. The scale bar indicates the nucleotide divergence per site

A maximum likelihood tree with 1000 bootstraps was produced from 49 concatenated, single copy genes predicted by BUSCO [27] from both *P. effusa* isolates and all published downy mildew assemblies available from NCBI, plus three diverse *Phytophthora* spp. and rooted with *Pythium ultimum* as the out-group (Fig. 5). Two downy mildew clades were evident. *P. effusa* was in the larger clade with *P. tabacina* and clusters with *Pseudoperonospora cubensis*, *H. arabidopsidis* and *Sclerospora graminicola*. The second downy mildew clade is made up of the three isolates of two *Plasmopara* spp., which were more closely related to *P. infestans* than to the other downy mildews.

### Mitochondria

The mitochondrial genomes of race 13 and 14 isolates were both 41,318 bp in size with a GC content of 22.8% (GenBank accessions MH142315 and MH325167, respectively). The coverage of the mitochondrial assemblies was 2081 and 1003x for R13 and R14, respectively. Sequences of both genomes were identical and had the same organization as *P. tabacina* (KT893455) with the exception of an inverted repeat (IR) that was present in *P. effusa*. Coding regions constituted 93.9% of the genome with 13.3% of this total representing hypothetical coding regions. A total of 35 known genes (encoding 18 respiratory chain proteins, 16 ribosomal proteins, and an import protein, *ymf16* of the *sec*Y-independent pathway), the *rnl* and *rns*, and 25 tRNA genes encoding for 19 amino acids were present. In addition, there were five hypothetical proteins (*ymf98*, *ymf99, ymf100, ymf101* and *orf32*) in common with other oomycete mitochondrial genomes [23, 37–42] and five putative ORFs that were unique to *P. effusa*. Four of the unique ORFs (*orf 181, orf131, orf201* and o*rf277*) were located between the *rns* and *cox2* genes (in the same location as the unique putative ORFs of *P. tabacina* but encoded in the opposite orientation) and another between the *atp1* and *nad5* genes (*orf209*). BLAST queries to GenBank identified no significant sequence similarities for *orf181, orf201,* and *orf209*; however, there was moderate sequence similarity of *orf131* and *orf 277* with putative ORFs in mitochondrial genomes of *P. tabacina* and *Phytophthora sojae* (DQ832717). In *orf131* of *P. effusa,* bases 287 to 393 were 75% identical to the 5′ end of *orf269a* of *P. tabacina*, while bases 7 to 262 were 69% identical to the 3′ end of this putative ORF. The 3′ end of *P. tabacina orf269a* (bases 323 to 743) shared similarity with putative ORFs from *P. sojae* with 85% identity to the spacer and 5′ end of *orf116*, while bases 59 to 320 are 89% identical to part of *orf101*. Bases 8–677 of *P. effusa orf277* were 73% identical to all but the last 100 bp of *P. tabacina orf269b* and bases 8–785 sharing 70% identity with the 5′ end of *P. tabacina orf290*.

A feature of the *P. effusa* mitochondrial genome that was not present in *P. tabacina* was the presence of an IR. The first arm of the IR was located between *orf201* and *orf277* with 382 bp of the 5′ end representing the 5′ end of *orf201*. The second arm of the IR was located between *atp1* and *orf209* with 382 bp of the 5′ end of the IR the 5′ end of *orf209*. The sequence for both arms of the IR is not a perfect match because there is a 2 bp deletion after base 667 in the first arm relative to the second repeat, hence the sizes are 872 bp and 874 bp, respectively. In addition to the IR there was also a 47 bp repeat present in two head to tail copies between *orf277* and *cox2*.

## Discussion

We present here two highly contiguous genome assemblies of an oomycete downy mildew pathogen. These assemblies of *P. effusa* contain 70 to 75% of the haploid genome size that is estimated to be 41 to 44 Mb by k-mer analysis. The missing component probably encodes repeat sequences that were not resolved from paired-end reads of short insert fragments, as the assemblies exceed the estimated size (~ 27 Mb) of the single copy portion of the genome. This assembly size is approximately half the smallest peak detected by flow cytometry and a quarter of the predominant peak, consistent with *P. effusa* being composed of a population of diploid and polyploid nuclei, as previously described for *Phytophthora* spp. [43, 44]. In comparison to assemblies of other downy mildews, these assemblies of *P. effusa* have fewer scaffolds and fewer contigs and have high BUSCO percent completeness scores indicating that they contain most of the gene space. The contig N_50_ of the *P. effusa* assemblies are similar to those of other downy mildews; however, the incorporation of long distance information from different technologies into assemblies of *H. arabidopsidis* [45] and *Plasmopara* spp. [3, 24, 25] resulted in higher scaffold N_50_s. The scaffold N_50_s are similar to those reported for *P. tabacina,* which were assembled using mate-pair reads [23] (Table 1).

The scaffolds of *P. effusa* are highly syntenic with those of *P. sojae* v3.0 (Fig. 1). *P. sojae* v3.0 is the most contiguous oomycete assembly available with 83 scaffolds and 99.12% of 82.6 Mb assembled into 31 scaffolds over 50 kb. The assemblies of *P. effusa* are more fragmented; 69% of R13 was encoded in 237 scaffolds over 50 kb, while 60% of R14 was contained in 214 scaffolds over 50 kb. The SyMap [31] plots (Fig. 1) provided little evidence for chimeric scaffolds in *P. effusa.* Furthermore, 177 R13 scaffolds and 167 R14 scaffolds could be ordered against 23 *P. sojae* scaffolds possibly indicative of pseudomolecules; pseudomolecules were not generated because of the potential for genome re-arrangements reported within the Peronosporales [46–50]. The generation of improved assemblies with chromosomal pseudomolecules awaits the future application of technologies that utilize long distance information [51].

The repertoires of annotated genes differed between *P. effusa* and *P. tabacina*. The assemblies of *P. tabacina* have a high BUSCO [27] duplication scores (~ 30%), indicating that these may not be high-quality consensus assemblies (i.e. the assembly is not representative of a single haplotype) and may have inflated the estimated number of gene models in *P. tabacina* compared to *P. effusa.* The BUSCO score of *P. tabacina* indicates that ~ 30% of the ~ 11,000 gene models may be duplicated; this is supported by the duplication of single copy regions in these assemblies detected by KAT [36] plots (Fig. 4b). If this is the case, there are ~ 8000 gene models in *P. tabacina*, similar to *P. effusa*. Interestingly, 4148 gene models were unique to one or the other isolate of *P. tabacina* (Fig. 2b) and 3095 genes were unique to *P. tabacina* when compared to other oomycetes (Fig. 2). The BUSCO duplication score for *P. effusa* was less than 0.5%; 1807 genes were unique to *P. effusa* when compared to other oomycetes (Fig. 2). Therefore, these isolates of *P. tabacina* may have more dispensable genes or not all genes were successfully assembled and annotated in both isolates of *P. tabacina*. This difference could be due to the isolates of *P. effusa* being genetically more similar to one another than the two isolates of *P. tabacina*, therefore having less opportunity to differentiate their gene repertoire. This difference in diversity is supported by both isolates of *P. effusa* having identical mitochondrial sequences, while those of the two *P. tabacina* isolates differed by seven SNPs, three indels and copy number of a 128 bp repeat [23]. Other oomycetes also have a higher unique gene content compared to *P. effusa* (Fig. 2) as well as good BUSCO scores, possibly indicating specialized sets of genes or misannotations in the other species. Our analysis was based on annotation developed using the transcriptomes of other oomycetes including *Hyaloperonospora*, *Bremia* and *Phytophthora* species [52–55]. RNAseq data for *P. effusa* may increase the number of unique or specialized genes identified in this species. Further genome and transcriptome sequencing of more isolates are required to characterize the pan genomes and extent of specialization in multiple oomycete species.

The effector repertoire of *P. effusa* is reduced in comparison to *Phytophthora* spp., similar to other downy mildews [3, 23, 45]. *P. effusa* has fewer RxLR motif encoding effectors, (~ 90) than *P. tabacina* (~ 165), although our analysis had the additional requirement of a degenerate EER motif or WY domain being encoded, which was not used for the predictions of *P. tabacina* [23]. More Crinklers (CRNs) were reported for *P. tabacina* (~ 130) than *P. effusa* (20; Table 2). *P. effusa* had a marginally higher incidence of genes models encoding putative pathogenicity associated domains but not RxLRs or CRNs (Table 3); ~ 1.7% of the gene models of each isolate encoded a putative pathogenicity domain vs. ~ 1.4% of the gene models for *P. tabacina* and *H. arabidopsidis*. *P. effusa* has a similar frequency of pathogenicity associated genes as *P. halstedii*, while over 2.5% of *Phytophthora* spp. gene models encode putative pathogenicity domains. Less than 3% of the predicted genes in *P. effusa* are implicated in pathogenicity.

Several Pfam [35] domains were found to be depleted in downy mildews, possibly reflecting adaptations to biotrophy (Table 6; Fig. 3). Eight transporter-associated Pfam domains were significantly depleted in *P. effusa* and all other downy mildews compared to *Phytophthora* spp. (Table 4) similar to previous reports [23, 45]. Many orthogroups containing gene models encoding these domains were not detected in downy mildews (Fig. 3, Table 6). The same analysis revealed the absence of orthogroups containing genes encoding carbohydrate binding or pathogenicity domains from all downy mildews (Fig. 3, Table 6). These observations are consistent with observations of the reduction in the frequency of pathogenicity associated proteins (Table 3) encoding kazal domains, pectate lyase, elicitin, and necrosis inducing proteins. Therefore, the repertoire of pathogenicity proteins seems to be consistently reduced in the biotrophic downy mildews.

Three of the four downy mildew species also had a reduction in calcium binding and flagella associated domains (Fig. 3, Table 6). The absence of flagella-associated domains is expected because *P. halstedii* is the only downy mildew species analyzed which produces zoospores [1, 49, 56]. The absence of genes encoding calcium associated domains (Fig. 3, Table 6) is consistent with the upregulation of one of the depleted domains (EF-hand) during sporangial development and cleavage during zoospore formation [55]. Genes encoding these domains would be under reduced selection in the absence of flagella. The missing genes encoding calcium-associated domains are good candidates for genes associated with zoospore biology.

Phylogenomics resolved two independent lineages of downy mildews (Fig. 5), consistent with other studies [2, 3], but not with those that infer a single origin [4]. Our topology placed *Peronospora* spp. in a clade that included *H. arabidopsidis*, *P. cubensis* and *S. graminicola*. This clade was separate from a clade that included the two species of *Plasmopara* spp. analyzed. The consistent loss of genes encoding transporter, phytopathogenicity, and carbohydrate-associated domains between these clades indicates that these genes are functional in the necrotrophic stages of *Phytophthora* spp. [57] and therefore have been lost in the biotrophic downy mildews.

The allele frequencies of *P. effusa* were not consistent with those of a regular diploid (Fig. 4c). Both isolates had alternative allele frequencies of ~ 0.33 in addition to the peak at 0.5 expected for a diploid. The absence of heterozygous 21-mers in *P. effusa* R14 (Fig. 4a) and the low frequency of SNPs detected when reads were mapped back to the assembly indicated that this isolate was largely homozygous. The frequency of SNPs was slightly higher in R13 though this was not proportional to the two clusters of 21-mers. Additionally, the 21-mers were not at the expected coverage for heterozygous loci (Fig. 4a). The majority of 21-mers from both of these clusters were present in the assembly (Fig. 4b), although the assembly size of R13 was not inflated relative to R14. If this was heterozygosity in R13 then half the 21-mers would be expected to be absent [36]. These results implied that few of the 21-mers were heterozygous in R13. The 21-mer profile of R13 cannot be explained by a mixture of isolates; shared 21-mers in a mixture of two isolates would be present in the highest coverage cluster and the lower coverage cluster would be made up of 21-mers differentiating the two. This is not what was observed (Fig. 4b). In addition, these 21-mers should not all be present in an assembly of a mixture of isolates because they would produce bubbles in the assembly graph that would be collapsed in a consensus assembly. The 21-mer profile of R13 indicates that this isolate has an ambiguous genomic architecture, in which a proportion of its genome has either been lost or duplicated. This was supported by the normalized read depth of genes (Fig. 4d), in which R13 had a wider spectrum of normalized read coverage than R14. While measurements by flow cytometry were not possible for these two isolates, later measurements of a separate isolate revealed that some nuclei were smaller at 80 Mb than the majority of 2C nuclei at 149 Mb (Additional file 1). The basis of the apparent variation in nuclear DNA size warrants further investigation to determine the prevalence of aneuploidy and polyploidy in *P. effusa* as has previously been documented for *Phytophthora spp.* [43, 44, 46, 58].

The mitochondrial genome of *P. effusa* is circular in orientation, approximately 41.3 kb in size and exhibited no sequence divergence between the genomes of race 13 and 14. The size is similar to other oomycetes (Table 8). The mitochondrial genome of *P. effusa* encodes the same common suite of genes, including the putative ORFs *ymf16, ymf98, ymf99, ymf100*, and *ymf101* as observed in the related taxa. There were also an additional four putative ORFs of unknown function encoded between *rns* and *cox2* and one putative ORF between the *atp1* and *nad5* genes that are unique to *P. effusa*, two of which share some level of sequence identity with a putative ORF in *P. tabacina* and *P. sojae*. The locations of the four species-specific putative ORFs between *rns* and *cox2* were the same as in *P. tabacina* and *P. cubensis*. Inverted repeats (IRs) have been observed in the mitochondrial genomes of the Peronosporomycete *Pythium* and Saprolegniomycetes *Saprolegnia, Achyla, Thraustotheca* and *Aphanomyces,* but these typically represent between 34 to 73% of the genome and encode the large and small ribosomal RNAs [37–39, 41, 59, 60]. While an IR is present in *P. effusa,* it is atypical for an oomycete because it is less than 900 bp and does not encode rRNA. These features are similar to the 1150 bp inverted repeat present in *P. ramorum* [40]. Recombination between the small IRs in *P. ramorum* generated isomers of the genome where the region between the repeats was also present in an inverted orientation [61] additional experimentation is needed with *P. effusa* to confirm if this is occurring in this taxon as well.

**Table 8:** Mitochondrial assembly statistics across the oomycetes

Family	Genus species	Isolate	Accession	Length	Inverted Repeat?	Inverted repeat length
Peronosporaceae	*Peronospora effusa*	R13		41.3 kb	Y	0.87 kb
		R14		41.3 kb	Y	0.87 kb
	*P. tabacina*	968-J2	NC028331	43 kb	N	
		968-S26	KT893456	43 kb	N	
	*Psuedoperonospora* *cubensis*		KT072718	38.6 kb	N	
	*Phytophthora andina*	EC3425	HM590419	37.9 kb	N	
	*P. infestans*	80029	AY894835	37.9 kb	N	
		15/99	AY898627	39.8 kb	N	
		94–52	AY898628	39.8 kb	N	
		WV4	NC002387	38 kb	N	
	*P. ipomoeae*	PIC99167	HM590420	37.9 kb	N	
	*P. mirabilis*	PIC99114	HM590421	37.8 kb	N	
	*P. nicotianae*		KY851301	37.6 kb	N	
	*P. phaseoli*	P18	HM590418	37.9 kb	N	
	*P. polonica*		KT946598	40.5 kb	N	
	*P. ramorum*	CBS 101553	EU427470	39.5 kb	Y	1.2 kb
		Pr-102	DQ832718	39.3 kb	Y	1.2 kb
	*P. sojae*	P6497	DQ832717	43.0 kb	N	
Pythiaceae	*Pythium insidiosum*	Pi-S	AP014838	55.0 kb	Y	18.3 kb
	*P. ultimum*	DAOM:BR114	GU138662	59.7 kb	Y	22 kb
Saprolegniaceae	*Achlya* *hypogyna*		KF226724	46.8 kb	Y	7.97 kb
	*Aphanomyces astaci*	AP03	KX405004	49.5 kb	Y	12.6 kb
	*A. invadans*	NJM9701	KX405005	49.1 kb	Y	12.4 kb
	*Saprolegnia* *ferax*	ATCC 36051	AY534144	46.9 kb	Y	8.6 kb
	*Thraustotheca* *clavata*		NC022179	47.4 kb	Y	9.4 kb

## Conclusions

We sequenced, assembled and annotated two isolates representing distinct races of *P. effusa*, the causal oomycete of spinach downy mildew. These assemblies are high quality (Table 1) and will serve as good references for this genus of over 500 species [1]. Approximately 8600 gene models were identified in each isolate that shared a high level of orthology between one another and with other oomycetes. Genes encoding domains associated with pathogenicity, transporters, and carbohydrate-binding were depleted across multiple downy mildews compared to *Phytophthora* spp. indicative of a parallel gene loss during the evolution of obligate biotrophy and genes associated with flagella were consistently absent in the non-flagellate downy mildews. These isolates of *P. effusa* were predominantly homozygous. High quality annotated assemblies of more isolates are required to resolve the complex genome architecture of *P. effusa.*

## Methods

### Phenotyping of isolates and DNA extraction

Genomic DNA samples for sequencing were obtained from two isolates of *P. effusa* collected from grower fields in Monterey County, California, in 2012 and 2013. The pathotypes of these isolates were determined by inoculation onto a differential set of spinach cultivars as previously described [8]. For both isolates, leaves of plants of a single cultivar showing heavy sporulation were collected and the spores were scraped off the leaf surface into water in a 50 ml tube as well as vortexed to remove additional sporangia. The suspension of the sporangia was transferred to a microfuge tube and spun at 21000x *g* for 1.5 min*.* The resulting pellet was washed in 1 ml of 95% ethanol, spun at 21000x *g* for 3 min and the pellet frozen at − 80 °C. Four hundred microliters of the Macherey-Nagel NucleoSpin Plant II kit (Düren, Germany) buffer PL1 and a single microfuge tube cap full of glass beads (Sigma G8772) was added and vortexed. The suspension was heat shocked at 65 °C with 10 μl RNase A solution, followed by another high-speed vortex. A 100 μl volume of chloroform was added, followed by a brief vortex and centrifugation for 5 min at 21000 *g*. The supernatant was added to a NucleoSpin® column (Machery-Nagel NucleoSpin Plant II kit), and the manufacturer’s plant DNA extraction protocol was followed.

### Flow cytometry

Flow cytometry was performed on sporulating and pre-sporulating spinach leaves mixed with 1 cm^2^ of young leaf tissue from *Oryza sativa* cv*.* Kitaake (2C = 867 Mb), which was sufficiently different from the genome size of *P. effusa* for use as the internal reference. The *O. sativa* 2C DNA content was determined by calibrating against nuclei from flower buds of *Arabidopsis thaliana* Col-0 which has a known absolute DNA content of 2C = 314 Mb [62]. Nuclei extraction and staining with propidium iodide was done using the Cystain PI absolute P kit (Sysmex, Lincolnshire, IL). Flow cytometry was done on a BD FACScan (Becton Dickinson, East Rutherford, NJ). For each measurement, 10,000 nuclei were assessed. Data was analyzed using FlowJo (Ashland, OR).

### Sequencing

Illumina TruSeq DNA libraries were prepared and sequenced at the Center for Genome Research & Biocomputing, Oregon State University (Corvallis, OR; http://cgrb.oregonstate.edu/core). DNA was quantified using a Qubit HS dsDNA assay (Invitrogen, Carlsbad, CA) and sheared by sonication followed by end repair, adenylation of 3′ ends, and adapter ligation. Fragments were purified by excision from an agarose gel, enriched by PCR, and the library was quantified with a Qubit HS dsDNA assay (Invitrogen). Sizing of the library was done using Agilent Bioanalyzer HS-DNA chip (Agilent Technologies, Waldbronn, Germany), with final quantification by qPCR using a KAPA Library quantification kit. The median library fragment sizes were 516 bp and 365 bp, for R13 and R14, respectively. Paired end libraries were prepared from genomic DNA of two *P. effusa* isolates (R13 and R14) and sequenced, 100 bp paired-end on an Illumina HiSeq 2000.

### Assembly

Reads were adapter and quality trimmed using BBMAP [63] and mapped to a reference containing bacterial and oomycete genomes available from NCBI, using BWA MEM, v0.7.12 [64], with flags -aC. Paired reads which mapped to oomycete genomes or failed to map to any organism were then advanced to assembly with MaSuRCA v2.3.2 [65]. Assemblies were done, specifying a JELLYFISH [30] size of 1 × 10^10^, in iterative k-mer steps of 10, ranging from 31 to 91, and 99 with additional assemblies performed at single step k-mer sizes flanking the highest scoring assemblies (measured on N_50_, assembly size, number of scaffolds > 100 kb, total number of scaffolds, BUSCO [27] score). The top five assemblies of each isolate were then positively filtered for oomycete scaffolds against NCBI nt, and negatively filtered for mitochondrial associated scaffolds with the mitochondrial assembly (produced as described below) using BLASTn [26]. Assemblies made up of scaffolds with a top BLASTn hit against oomycete scaffolds and with a minimum scaffold size of 1 kb, were then merged in a step-wise manner from lowest scaffold number to highest using Quickmerge [66]. Repeat libraries were generated with RepeatModeler [32], assemblies soft-masked with RepeatMasker [67] and secondary haplotypes collapsed, first with Redundans [28], then Haplomerger2 [29]. Final assembly statistics were obtained using BBMAP [63] and compared to previously assembled genomes. Completeness statistics were obtained with BUSCO.

Assemblies were aligned against one another with NUCmer [68] (−l 100), and both were independently aligned against *P. sojae* v3.0 with PROmer [68] (−l 30). These were ran and visualized as part of SyMap [31], with a minimum dot requirement of 5 and allowing merged blocks. REAPR [69] summary scores of the final and intermediate assemblies were calculated as previous [70]. REAPR summary scores of both isolates increased as post-assembly processing was performed on each isolate with the final draft assemblies presented here having the highest score (Additional files 2 and 14).

### Annotation

The annotation workflow is depicted in Additional file 8. *Ab initio* annotation was performed with SNAP [33] as part of the MAKER pipeline [34]. Initial predictions were generated with MAKER from all oomycete EST and protein data available on NCBI (est2genome = 1, protein2genome = 1) masking with the above generated repeat library. These predictions were used to produce a HMM using SNAP default settings (fathom, forge, hmm-assembler.pl) and then bootstrapped through subsequent rounds of MAKER, the first using the est2genome and protein2genome evidence for prediction, with subsequent runs turning this off to use only the SNAP HMM for gene prediction (est2genome = 0, protein2genome = 0). All maker runs surveyed single exon proteins with a minimum nucleotide length of 240 being considered (single_exon = 1, single_length = 240). The optimal run was identified through comparative analysis of alternative predictions, namely runs were scored for BLAST [26] hits to the Oomycete training protein sequences, % orthology detected with the Oomycete training database as detected by OrthoFinder [71] and average e-value of Pfam [35] domains detected with InterProScan [72] with a value under 1e^− 5^.

Additional putative effector gene models were identified from all ORFs predicted from the genome over 80 residues in length with no missing data. All ORFs were surveyed for secretion signals using SignalP v4.1 (−u 0.34) and independently with SignalP v3.0 (default settings) [73, 74]. ORFs were considered secreted if a positive result was obtained through either or both approaches and no trans-membrane domains were detected by SignalP v4.1. Further filtering, removing ORFs targeted to the mitochondria was performed using TargetP [75]. All ORFs predicted (i.e. regardless of signal peptide prediction) were surveyed for Crinklers (CRN) and WY repeats using hidden Markov models (HMMs) with HMMER v3.1b1 [76]. The CRN HMM was produced with hmmsearch, inputting an alignment of all labelled *Phytophthora* spp. CRNs from NCBI. The WY HMM was described previously [77, 78]. Predicted secreted ORFs were surveyed for RxLR motifs and previously described/queried degenerate [GHQ]xLR or RxL[GKQ] motifs [53, 79–82] linked with either degenerate [DE][DE][ER] motif or a WY repeat [78]. Predicted effectors were integrated into the Maker produced GFF file by identifying over-lapping gene models on the same strand with BEDTools [83]. Gene models were characterized as to whether they shared either or both start and stop codon positions.

Candidate effectors at unique genomic loci were further curated to ensure that they did not overlap or neighbor one another within 1 kb on the same strand. In instances when not the case, these models were manually refined, with the aid of BLAST [26], to produce multi-exonic RxLR-(EER)-WY models [84, 85].

The final predicted protein sequence was then generated from the GFF using GAG and putative effectors were re-identified on the entire annotation set to obtain final putative effector counts, which include those independently predicted by MAKER [34]. Conserved functional domains, including pathogenicity associated domains, of the final gene models were identified with InterProScan [72] and putative gene model names were assigned through stringent BLAST to the UniProt reference database [86]. These features were added to the NCBI table file with Annie and GAG [87, 88].

### Comparative genomics

De novo identification of genes encoding pathogenicity associated domains for *P. effusa,* other downy mildew and *Phytophthora* species was performed by running InterProScan on all available gene models. Gene models were inferred to contain a pathogenicity associated domain if such a domain was identified by CDD, Gene3d, PANTHER, Pfam, PRINTS, ProDom, ProSitePatterns, ProSiteProfiles, SMART or SUPERFAMILY [35, 72, 89–94]. The total frequency of gene models encoding pathogenicity associated domains was calculated as the number of unique gene models identified with any of the specified domains divided by the total number of gene models surveyed. T-tests were performed on the frequency of models containing each domain and on the total frequency of pathogenicity associated gene models, segregating the results as downy mildew vs. *Phytophthora* and *Peronospora* spp., and *H. arabidopsidis* vs. *Phytophthora* spp. and *P. halstedii*. Chi-squared testing was performed on Pfam [35] domains detected across all seven species. In this test, a protein was counted once for each unique domain it encoded (i.e. it was not weighted if it encoded multiple of a single domain type). Expected scores were weighted for the number of proteins detected as having Pfam domains in each species. Tests were performed on all domains as *P. effusa* vs. all, *Peronospora* spp. vs. all, *Peronospora* spp. and *H. arabidopsidis* vs. all, and downy mildews vs. *Phytophthora* spp. A Bonferroni-adjusted *p*-value was calculated based on the number of domains tested, with Pfam domain scoring below this p-value manually investigated. Instances where an over-representation of domains in *P. tabacina* appeared to score a significant result were investigated, owing to the high duplication in the *P. tabacina* assemblies detected in this study.

Orthogroups were inferred using OrthoFinder [71]. First gene models of *P. effusa* isolates were compared in a two-way analysis. Proteins detected as absent between isolates were investigated by mapping reads of one isolate to the other isolates assembly using BWA MEM v0.7.12 [64] and calling SNPs/Indels with SAMtools mpileup v0.1.18 [95]. For comparison, the same was replicated on the two *P. tabacina* isolates. These isolates were then combined into a set for their respective species and a seven-way comparison was performed, including *H. arabidopsidis*, *P. infestans, P. ramorum*, *P. sojae* and *P. halstedii*. Intersects were visualized with upsetR [96] and orthology coefficients (OC) were calculated as OC = (C/T_1_) x (C/T_2_), where C is the number of overlapping orthologous groups, T_1_ is the total number of orthologous groups identified in sample 1 and T_2_ is the total number of orthologous groups identified in sample 2. Orthogroups which contained gene models encoding Pfam domains, inferred as under-represented by the Chi-squared analysis, in downy mildew assemblies were identified and analyzed for presence/absence of each species. An orthogroup was considered single copy if only 0–1 models were present for each *Phytophthora* spp., *P. halstedii* and *H. arabidopsidis* and 0–2 models detected for *P. tabacina* and *P. effusa*, owing to a) two isolates being used in the analysis for both *Peronospora* spp. and b) the high level of duplication detected in *P. tabacina* assemblies. Finally, orthogroups containing previously identified *P. infestans* nitrogen and sulphur assimilation enzymes [45] were identified to obtain orthologs of these enzymes in downy mildew species.

### K-mer and read analysis

JELLYFISH [30] 21-mer hashes for individual read files were generated and histograms were obtained to estimate the genome size of both isolates. These were plotted with R [97] to obtain the 21-mer boundaries of single copy regions of the genome. K-mer based genome size estimates were calculated by summing the results of k-mer density multiplied by its frequency. Estimates of the size of the single copy portion of the genome were produced by limiting this calculations to k-mers with densities between the limits of the k-mer peak profiles (R13; 82–340, R14; 142–560; Additional file 13). Hashes were visually inspected through KAT density plots [36], for both *P. effusa* isolates and comparatively for two previously sequenced *P. tabacina* isolates [23]. Hashes generated from pairs of read files were also compared to assembly hashes generated from resulting isolate assemblies and visually inspected through KAT spectra-cn plots.

Heterozygosity of each isolate was estimated by calling SNPs from reads mapped back to either assembly, using SAMtools v0.1.18 mpileup [95] and calculating the number of heterozygous sites, defined as those with an allele frequency between 0.2 and 0.8. Plotting the frequency of the alternative allele of heterozygous SNPs was performed by extracting the number of reads supporting the reference and alternative allele at each bi-allelic SNP site and obtaining the frequency of reads supporting the alternative allele and binned to the nearest hundredth. For SNP sites to be counted they had to be covered by a minimum of 50 reads. The number of SNPs per bin were summed and plotted in R [97]. Variant call files containing the SNP coordinates were intersected with genic lines of the annotated gff using BEDTools [83] to obtain SNP counts within genic regions and the allele frequency plots were produced as above. Read coverage per gene was calculated by obtaining the number of reads per genic locus, using BEDTools multicov [83] requiring the assembly file and an indexed binary alignment map file, multiplied by the read length, divided by the length of the gene. The coverage was normalized for each isolate (calculated from a BEDTools genomecov plot) and plotted in R using ggplot2 [98] applying a multiplicate bandwidth adjustment of ten.

### Phylogenomics

Phylogenetics of BUSCO [27] orthologs across a panel of published, publicly available downy mildew assemblies was carried out with select, high quality *Phytophthora* assemblies and rooted with *Pythium ultimum*. BUSCO was run independently on every assembly to be surveyed and single copy orthologs from each were identified. Amino acid sequences of orthologs present in all isolates were then aligned independently using MAFFT v7.123b [99] (Additional file 15). Alignments were concatenated and RAxML v8.0.26 [100] was run with 1000 bootstraps and the PROTGAMMAAUTO substitution model. The resulting tree was rooted with *P. ultimum* and visualized in Geneious and labels were manually placed to improve legibility.

### Mitochondrial assembly and annotation

Contigs from a de novo genomic assembly in CLC Genomics Workbench (v8; Qiagen, Redwood City, CA) were identified as mitochondrial due to sequence similarity with *P. tabacina* mitochondrial sequences (KT893455) by BLAST analysis. These were used as templates for further assembly with SeqMan NGen (v4.1.2, DNASTAR, Madison, WI, USA). The resulting assemblies were evaluated for uniformity and depth of coverage. Contigs were broken when gaps/low coverage or inconsistencies were observed and the set of smaller contigs reassembled using the small templated assembly option of SeqMan NGen to extend the ends of the contigs and the close gaps. ORFs were predicted and annotated with DS Gene v1.5 (Accelrys, San Diego, CA) using the universal genetic code with confirmation of gene identities using BLAST [26] analysis against mitochondrial genome sequences published for *P. tabacina*, *Pythium*, and *Phytophthora* spp. [23, 38, 40] tRNA coding regions were placed using tRNAscan-SE v1.3.1 [101].

## Additional files

Additional file 1: Flow cytometry measurements of a *P. effusa* isolate. (PDF 305 kb)

Additional file 2: Intermediate assembly statistics and REAPR scores. (XLSX 24 kb)

Additional file 3: JELLYFISH histogram outputs and line plots. (7Z 171 kb)

Additional file 4: RepeatModeler summary of outputs. (XLSX 8 kb)

Additional file 5: Long Terminal Repeat plots. Long terminal repeats are plotted as previously described [23]. The top panel show the distribution of insertion estimates for each LTR family, the number to the right reports the number of members in that family. The bottom panel estimates the time from initial insertion of the major LTR super-families; retrotransposon LTR Gypsy (RLG) and retrotransposon LTR Copia (RLC). (JPEG 243 kb)

Additional file 6: Intermediate annotation results. Summary of maker runs bootstrapping SNAP hidden markov model. (XLSX 13 kb)

Additional file 7: Annotation pipeline. Workflow overview for the annotation of *P. effusa*. (PNG 242 kb)

Additional file 8: ORF annotation results. Summary of the results from hmm search and regular expression string searches when applied to all open reading frames of both *P. effusa* isolates. (XLSX 8 kb)

Additional file 9: Manual ORF curation. Image summarizing the initial ORFs predicted, manually curated exon structure, and RxLR, EER and WY locations along genomic fragments of both *P. effusa* isolates. Three were annotated in each isolate. The ORFs are not aligned. (PNG 123 kb)

Additional file 10: Significant chi-squared results. All significant Chi squared results detected, when comparing the occurrence of domains encoded by *P. effusa*, *P. tabacina*, *H. arabidopsidis*, *P. halstedii*, *P. ramorum*, *P. sojae* and *P. infestans*. (XLSX 32 kb)

Additional file 11: Read alignment summary of isolate specific genes. Details genes which were found to contain inter-isolate indels or SNPs which affected either the start or stop codon position of the protein. To query P.effusa proteins in GenBank, substitute PeffR13 with DD237, PeffR14 with DD238 and remove the -RA suffix. (XLSX 17 kb)

Additional file 12: Orthogroups unique to *Peronospora* spp. Details sequence names of all proteins inferred as being orthologous only between *P. effusa* and *P. tabacina*. To query P. effusa proteins in GenBank, substitute PeffR13 with DD237, PeffR14 with DD238 and remove the -RA suffix. (XLSX 10 kb)

Additional file 13: Orthogroup overview. Listing all the orthogroups identified and the genes they contain, which were plotted in Fig. 4. To query P. effusa proteins in GenBank, substitute PeffR13 with DD237, PeffR14 with DD238 and remove the -RA suffix. (7Z 21 kb)

Additional file 14: REAPR scores. Summarizing the REAPR scores reported in Additional file 1. (JPEG 89 kb)

Additional file 15: BUSCO alignments used to create Fig. 5. The alignments are not concatenated. Note: .7z files can be accessed by downloading 7zip. This software is free and available at https://www.7-zip.org/. (7Z 50 kb)

## Abbreviations

bp: Base-pair
CRN: Crinkler
*H. arabidopsidis*: *Hyaloperonospora arabidopsidis*
HMM: Hidden Markov model
IR: Inverted repeat
kb: Kilo-base-pair
LTR: Long terminal repeat
Mb: Mega-base-pair
ml: Milliliter
ORF: Open reading framw
*P. cubensis*: *Pseudoperonospora cubensis*
*P. effusa*: *Peronospora effusa*
*P. farinosa*: *Peronospora farinosa*
*P. halstedii*: *Plasmopara halstedii*
*P. infestans*: *Phytophthora infestans*
*P. ramorum*: *Phytophthora ramorum*
*P. sojae*: *Phytophthora sojae*
*P. tabacina*: *Peronospora tabacina*
*P. ultimum*: *Pythium ultimum*
R: Race
R13: Race 13
R14: Race 14
*S. graminicola*: *Sclerospora graminicola*
*S. oleracea*: *Spinacia oleracea*
SNPs: Single nucleotide polymorphisms
*spp.*: Species
μl: Microliter
