# Supplementary material for: Comparative genomics of downy mildews reveals potential adaptations to biotrophy
Source: BMC Genomics. 2018 Nov 29;19:851. doi: 10.1186/s12864-018-5214-8 (PMC6264045; doi:10.1186/s12864-018-5214-8)
Supplement: Supplementary file 1 — Flow cytometry measurements of a P. effusa isolate. (PDF 305 kb) [file 12864_2018_5214_MOESM1_ESM.pdf]

# Additional File 1 – Flow cytometry of *P. effusa*

Table A1.1. Calculated nuclear content of each peak (see Figure A1.1. for an example). Pe2 contains the most nuclei and is used to estimate the genome size of *P. effusa.* Replications available in Table A1.2.

| | Average (Mb) | Stdev (Mb) | CV | Count |
|---|---|---|---|---|
| Pe1 | 80.1 | 1.9 | 2.4 | 3 |
| Pe2 | 148.6 | 9.0 | 6.1 | 8 |
| Pe3 | 300.2 | 18.5 | 6.2 | 8 |

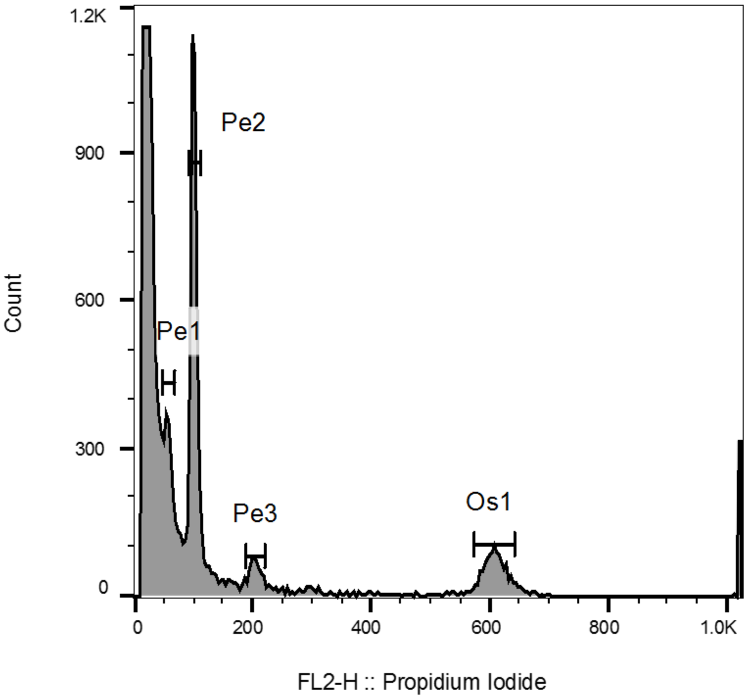


Figure A1.1. Flow cytometry propidium iodide histogram of *P. effusa* (first three peaks) relative to *Oryza sativae* (867 Mb). In the above figure Pe1 = 78.5 Mb, Pe2 = 146.5 Mb, Pe3 = 291 Mb, Os=867 Mb.

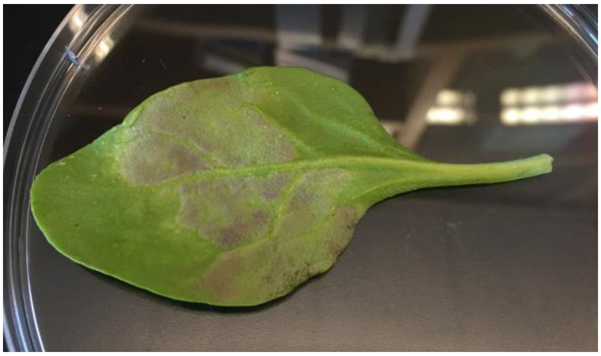

Figure A1.2. Spinach leaf infected with *P. effusa*, seen as grey.

Table A1.2. FL2-H measurement of *P. effusa* and *O. sativa* nuclei and calculated nuclear content. Pe1 peak is not always present. It is likely that it is masked by the cell debris to the left.

| Replication | date | Pe1 | Pe2 | Pe3 | Os1 | Pe1 (Mb) | Pe2 (Mb) | Pe3 (Mb) |
|---|---|---|---|---|---|---|---|---|
| 1 | 1/15/2016 | n/a | 56.5 | 114 | 290 | | 168.9 | 340.8 |
| 2 | 1/15/2016 | n/a | 44.5 | 90 | 274 | | 140.8 | 284.8 |
| 3 | 1/15/2016 | 26 | 48.5 | 96.4 | 287 | 78.5 | 146.5 | 291.2 |
| 4 | 1/15/2016 | n/a | 52 | 106 | 294 | | 153.3 | 312.6 |
| 5 | 1/15/2016 | n/a | 48.4 | 98.8 | 288 | | 145.7 | 297.4 |
| 6 | 1/25/2016 | 31.8 | 56.6 | 115 | 347 | 79.5 | 141.4 | 287.3 |
| 7 | 1/25/2016 | 57.6 | 102 | 204 | 607 | 82.3 | 145.7 | 291.4 |
| 8 | 1/25/2016 | n/a | 65.4 | 132 | 387 | | 146.5 | 295.7 |
