# Supplementary figures and images for: Comparative genomics of downy mildews reveals potential adaptations to biotrophy
Source: BMC Genomics. 2018 Nov 29;19:851. doi: 10.1186/s12864-018-5214-8 (PMC6264045; doi:10.1186/s12864-018-5214-8)

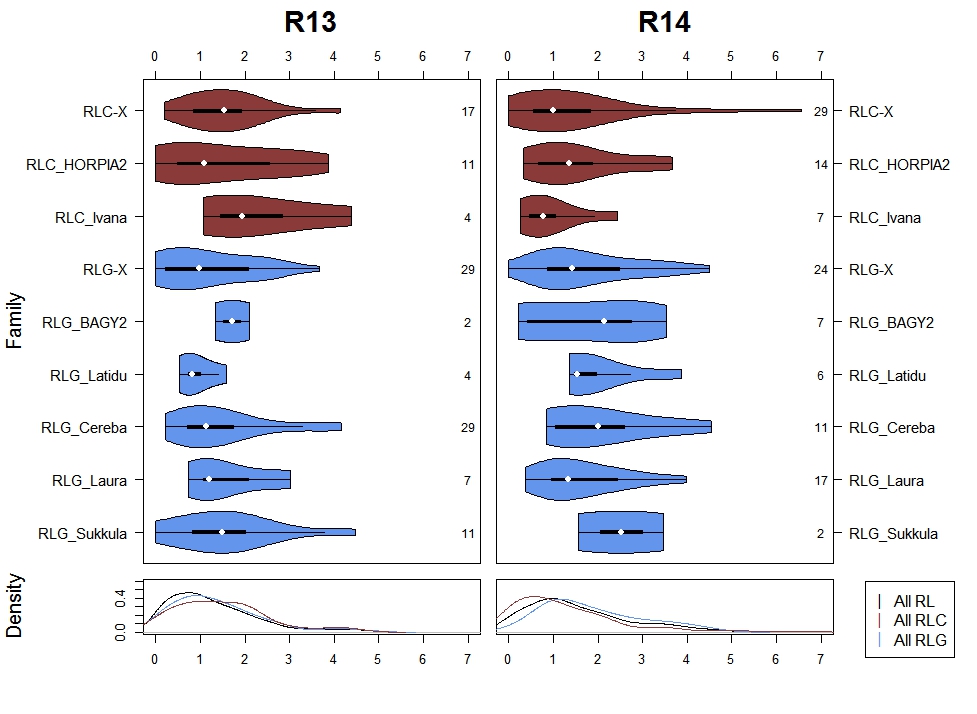

Supplement: Supplementary file 5 — Long Terminal Repeat plots. Long terminal repeats are plotted as previously described [23]. The top panel show the distribution of insertion estimates for each LTR family, the number to the right reports the number of members in that family. The bottom panel estimates the time from initial insertion of the major LTR super-families; retrotransposon LTR Gypsy (RLG) and retrotransposon LTR Copia (RLC). (JPEG 243 kb) [file 12864_2018_5214_MOESM5_ESM.jpeg]

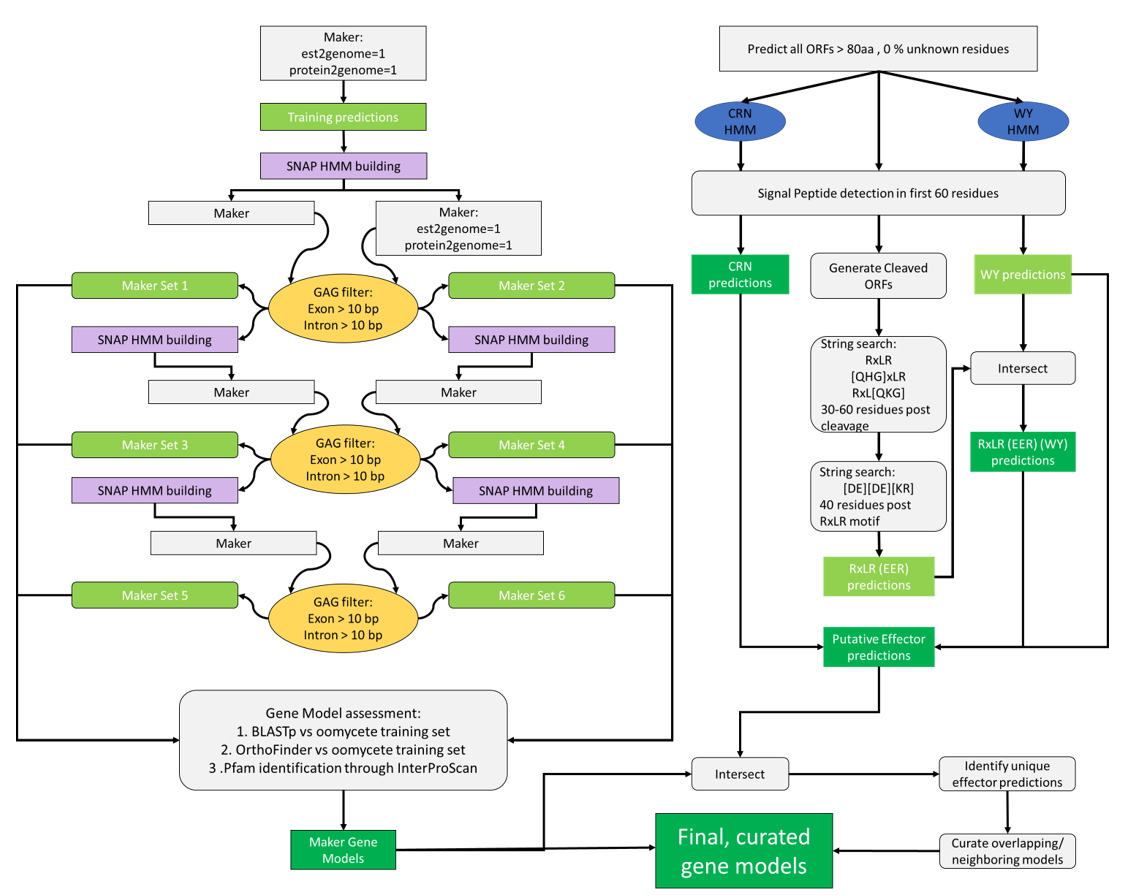

Supplement: Supplementary file 7 — Annotation pipeline. Workflow overview for the annotation of P. effusa. (PNG 242 kb) [file 12864_2018_5214_MOESM7_ESM.png]

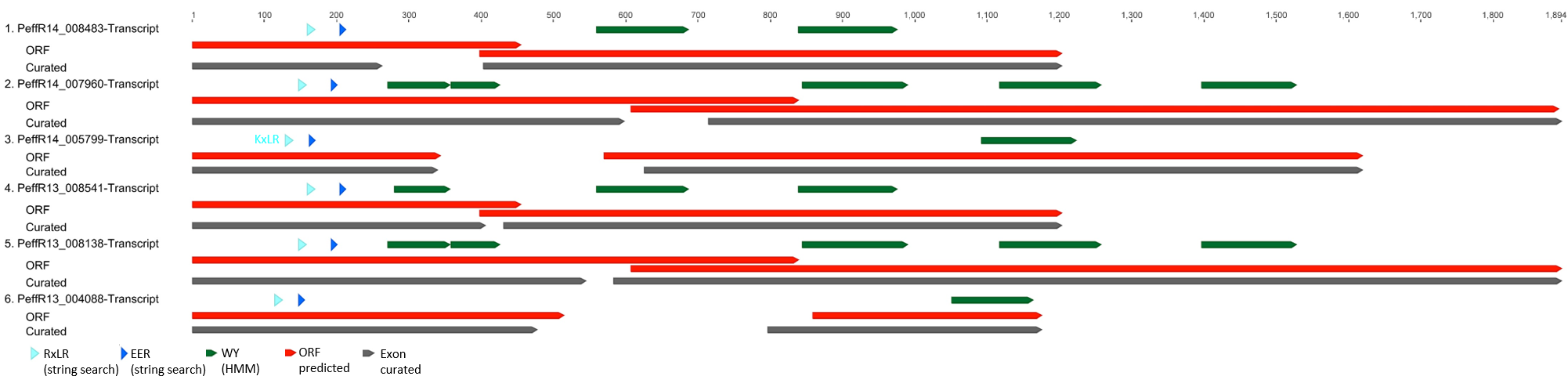

Supplement: Supplementary file 9 — Manual ORF curation. Image summarizing the initial ORFs predicted, manually curated exon structure, and RxLR, EER and WY locations along genomic fragments of both P. effusa isolates. Three were annotated in each isolate. The ORFs are not aligned. (PNG 123 kb) [file 12864_2018_5214_MOESM9_ESM.png]

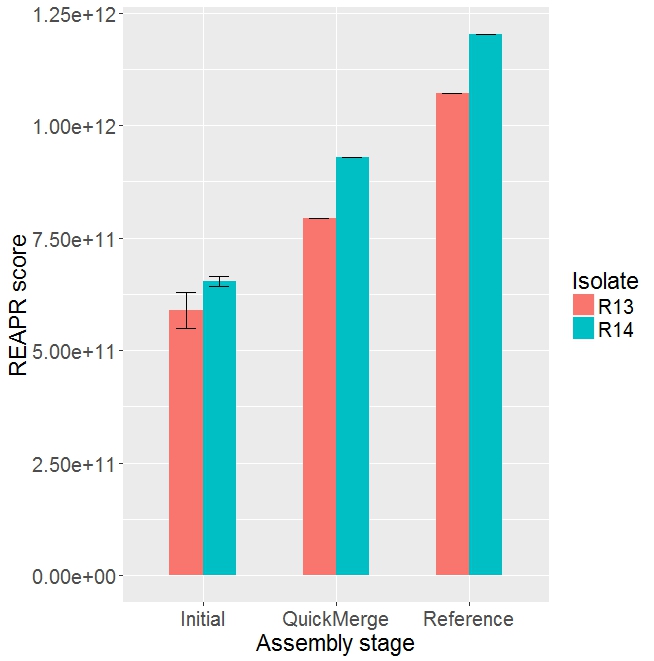

Supplement: Supplementary file 14 — REAPR scores. Summarizing the REAPR scores reported in Additional file 1. (JPEG 89 kb) [file 12864_2018_5214_MOESM14_ESM.jpeg]
